# Dynamic changes in eIF4F-mRNA interactions revealed by global analyses of environmental stress responses

**DOI:** 10.1186/s13059-017-1338-4

**Published:** 2017-10-27

**Authors:** Joseph L. Costello, Christopher J. Kershaw, Lydia M. Castelli, David Talavera, William Rowe, Paul F. G. Sims, Mark P. Ashe, Christopher M. Grant, Simon J. Hubbard, Graham D. Pavitt

**Affiliations:** 1 Division of Molecular and Cellular Function, School of Biological Sciences, Faculty of Biology Medicine and Health, Manchester Academic Health Science Centre, The University of Manchester, Manchester, M13 9PT UK; 2 Division of Evolution and Genomic Sciences, School of Biological Sciences, Faculty of Biology Medicine and Health, Manchester Academic Health Science Centre, The University of Manchester, Manchester, M13 9PT UK; 3 Division of Cardiovascular Sciences, School of Medicine, Faculty of Biology Medicine and Health, Manchester Academic Health Science Centre, The University of Manchester, Manchester, M13 9PT UK; 4 Manchester Institute of Biotechnology (MIB), The University of Manchester, 131 Princess Street, Manchester, M1 7DN UK; 5 Present address: Biosciences, College of Life and Environmental Sciences, Geoffrey Pope Building, University of Exeter, Stocker Road, Exeter, EX4 4QD UK; 6 Present address: Sheffield Institute for Translational Neuroscience, The University of Sheffield, 385a Glossop Road, Sheffield, S10 2HQ UK; 7 Present address: Department of Chemistry, Loughborough University, Epinal Way, Loughborough, Leicestershire LE11 3TU UK

**Keywords:** eIF4F, Translational control, Yeast, Stress regulation of gene expression

## Abstract

**Background:**

Translation factors eIF4E and eIF4G form eIF4F, which interacts with the messenger RNA (mRNA) 5′ cap to promote ribosome recruitment and translation initiation. Variations in the association of eIF4F with individual mRNAs likely contribute to differences in translation initiation frequencies between mRNAs. As translation initiation is globally reprogrammed by environmental stresses, we were interested in determining whether eIF4F interactions with individual mRNAs are reprogrammed and how this may contribute to global environmental stress responses.

**Results:**

Using a tagged-factor protein capture and RNA-sequencing (RNA-seq) approach, we have assessed how mRNA associations with eIF4E, eIF4G1 and eIF4G2 change globally in response to three defined stresses that each cause a rapid attenuation of protein synthesis: oxidative stress induced by hydrogen peroxide and nutrient stresses caused by amino acid or glucose withdrawal. We find that acute stress leads to dynamic and unexpected changes in eIF4F–mRNA interactions that are shared among each factor and across the stresses imposed. eIF4F–mRNA interactions stabilised by stress are predominantly associated with translational repression, while more actively initiating mRNAs become relatively depleted for eIF4F. Simultaneously, other mRNAs are insulated from these stress-induced changes in eIF4F association.

**Conclusion:**

Dynamic eIF4F–mRNA interaction changes are part of a coordinated early translational control response shared across environmental stresses. Our data are compatible with a model where multiple mRNA closed-loop complexes form with differing stability. Hence, unexpectedly, in the absence of other stabilising factors, rapid translation initiation on mRNAs correlates with less stable eIF4F interactions.

**Electronic supplementary material:**

The online version of this article (doi:10.1186/s13059-017-1338-4) contains supplementary material, which is available to authorized users.

## Background

Eukaryotic messenger RNAs (mRNAs) bear a 5′ m7GpppN cap structure that is added co-transcriptionally in the nucleus and is important for their translation and stability in the cytoplasm. eIF4E is the major cytoplasmic cap-binding protein that, together with eIF4G, plays important roles enhancing translation rates via recruiting 40S ribosomes and associated factors for translation initiation events. eIF4G binds to eIF4E via conserved regions on the surfaces of each factor [1, 2]. The poly A tail at the 3′ UTR also enhances translation rates, acting synergistically with the 5′ cap [3]. Multiple lines of evidence support the formation of an mRNA ‘closed loop’ wherein the poly A binding protein interacts with both the poly A tail and eIF4G that in turn simultaneously binds to 5′ cap-bound eIF4E. Such a structure has been demonstrated to circularise mRNAs and is proposed to explain the enhanced translation rates of capped and polyadenylated mRNAs. However, the mechanisms of translational enhancement are not yet clear [4, 5].

eIF4E and eIF4G function at the 5′ cap to help recruit active eIF2-ternary complex (TC)–bound small ribosome subunits (40S) to the mRNA 5′ end. This complex contains additional translation factors including eIF1, eIF1A, eIF3 and eIF5 and is termed the 43S pre-initiation complex [4]. Interactions between eIF4G and eIF3 or eIF5 serve as a bridge to the 40S subunit [6, 7]. 40S recruitment is also facilitated by the RNA helicase eIF4A, which can unwind mRNA secondary structures close to the 5′ cap. By promoting this cap-dependent initiation pathway for ribosome recruitment, the eIF4F components may also help prevent aberrant competing pathways, such as eIF3-led mRNA recruitment [8].

Translation initiation occurs at different rates on individual mRNAs and contributes to the discrepancies seen between protein and mRNA levels when measured globally [9–11]. Mechanistically, how the dynamics of translation factor binding to individual mRNAs influences initiation frequencies is not yet understood, as very few mRNAs and/or factors have been studied intensely. However, a global computational analysis suggested that the time taken for an individual initiation event can vary over 100-fold between individual yeast mRNAs (in the range of approximately 1 s to > 130 s) [12]. This is in agreement with multiple studies indicating that initiation is rate-limiting for the overall translation process [4].

We recently employed an RNA-immune precipitation and next-generation sequencing approach (RIP-seq) to quantify the abundance of mRNAs associating with different translation factors and RNA-binding proteins in budding yeast, making use of isogenic strains bearing individual TAP-tagged proteins [13–15]. We examined interactions between mRNAs and eIF4E, the two isoforms of eIF4G (4G1 and 4G2), Pab1 and the two yeast eIF4E-binding proteins (4E-BPs) Caf20 and Eap1 that can compete with eIF4G for binding to eIF4E [13]. While there was relatively little variation in the enrichment of mRNAs with Pab1, which can bind multiple times to the poly A tail, there were marked differences in the association of the other proteins examined and some results were unexpected. We found that eIF4E, eIF4G1 and eIF4G2 were co-enriched or depleted across many hundreds of mRNAs, such that there were almost no differences in the enrichment patterns for all three proteins across thousands of mRNAs. Similarly, the 4E-BPs shared similar enrichment patterns to each other that were distinct from the eIF4F proteins. By clustering the mRNAs, we grouped nearly 3000 mRNAs into four broad mRNA classes that were either relatively co-enriched (Groups III and IV) or co-depleted (Groups I and II) for eIF4E and eIF4G and also either relatively co-enriched (Groups II and IV) or co-depleted (Groups I and III) for the 4E-BPs [13]. In accord with predictions from the closed-loop model, Group III mRNAs (bound by eIF4E and eIF4G, but depleted for the 4E-BPs) encode abundant proteins and have high translation efficiencies (TEs) as inferred from ribosome footprinting studies, while the 4E-BP enriched mRNA classes had lower TEs [13].

Subsequent analyses showed that Group III mRNAs, also called the ‘strong closed-loop’ set, had short ORFs [16, 17] and that they were also preferentially bound by ribosomes bearing the Asc1/RACK1 protein [17] leading to the idea that Asc1/RACK1 helps direct ribosomes to bind and promote translation on Group III mRNAs with short ORFs [5, 17]. Further genome-wide analyses indicated that Group III mRNAs were translated largely independent of eIFs 4A, 4B and Ded1, as translation of these mRNAs was relatively unaffected by mutants inactivating each factor [18]. Two unexpected groups were Groups I and II. Group II was depleted for eIF4F and enriched for the 4E-BPs. An explanation for this observation was found subsequently, as both yeast 4E-BPs were found to interact with translating ribosomes engaged with specific mRNAs independently of 4E-BP–eIF4E interactions [16]. In contrast, Group I mRNAs were relatively depleted for closed-loop proteins but have high TEs and encode abundant proteins. This group included mRNAs encoding many glycolytic enzymes. It was suggested that these mRNAs may recruit ribosomes via an alternative mechanism, possibly involving Pab1. Indeed, earlier observations suggested that Pab1 can stimulate translation by multiple means [3, 19]. In addition, there is evidence that eIF3 and eIF2 can promote binding of some mRNAs to 40S ribosomes independently of eIF4G [20]. However, the mechanism of mRNA selection operating on Group I RNAs remains to be determined.

A range of acute stresses causes widespread reprogramming of translation initiation in different cell types [21, 22]. We, and others, have studied environmental stress responses in yeast on a global scale [22]. Relevant to this present study are glucose and amino acid nutritional starvations as well as oxidative stress induced by hydrogen peroxide addition. Both amino acid and peroxide stresses promote phosphorylation of eIF2. This impairs translation initiation globally by inhibiting the activity of eIF2B, the guanine nucleotide exchange factor that normally activates eIF2 to promote TC formation [23–25]. Thus, eIF2 phosphorylation lowers global translation by interfering with TC formation. Both stresses also activate *GCN4* translation (a transcriptional activator of amino acid biosynthetic genes) as upstream ORFs inversely couple eIF2B activity and *GCN4* translation levels [4]. Thus, translational control activates stress-responsive regulatory networks. Despite these common events, each stress impacts distinctly on the translation of some individual mRNAs, indicating that other controlling elements are important to both repress bulk translation and promote translation of specific stress-responsive mRNAs to promote recovery [26–29]. In contrast, short-term glucose withdrawal does not promote phosphorylation of eIF2 and translational repression therefore operates via a distinct mechanism [30]. It was shown that loss of eIF4A from translating ribosomes occurs early following glucose removal [31] and so inhibition of eIF4A activity may contribute to glucose-mediated translational repression. Following glucose depletion, many mRNAs become unstable [32], while others are localised within cytoplasmic granules called P bodies that contain both eIF4E and RNA-decay proteins. In addition, ‘stress’ granules (also termed ‘EGP bodies’) form that lack RNA-decay proteins but contain mRNAs and some translation factors including eIF4E, eIF4G and Pab1 [33–35]. mRNAs translationally activated following glucose starvation include pentose-phosphate pathway mRNAs [31]. It was also found that heat shock mRNAs transcribed from promoters bound by Hsf1 were translationally activated rather than being localised to P bodies or stress granules [36].

As we unexpectedly found that eIF4E and eIF4G were differently associated with mRNAs [13], and because the role of these 5′ cap associated factors in the translational responses to stress in yeast had not been evaluated, we have now investigated how eIF4E/G1/G2–mRNA interactions are changed in response to these three acute stresses that reprogram translation. Following a previously used RIP-seq approach, [13], we find that each stress does alter the pattern of association of mRNAs with each factor in a coordinated way. Many of the changes observed are not stress-specific, suggesting that there is a common early general stress response that alters eIF4F–mRNA interactions. Our analyses indicate that the direction of change in eIF4F interactions with many mRNAs opposes both changes in transcript levels and changes in ribosome occupancy following stress. We find that the Group I mRNAs, as described above, are particularly sensitive to stress and interpret our data as compatible with a model where multiple mRNA closed-loop complexes can form with differing stability.

## Results

### Stresses inhibit translation initiation but do not promote large changes in closed-loop factor protein–protein interactions

**Fig. 1:**
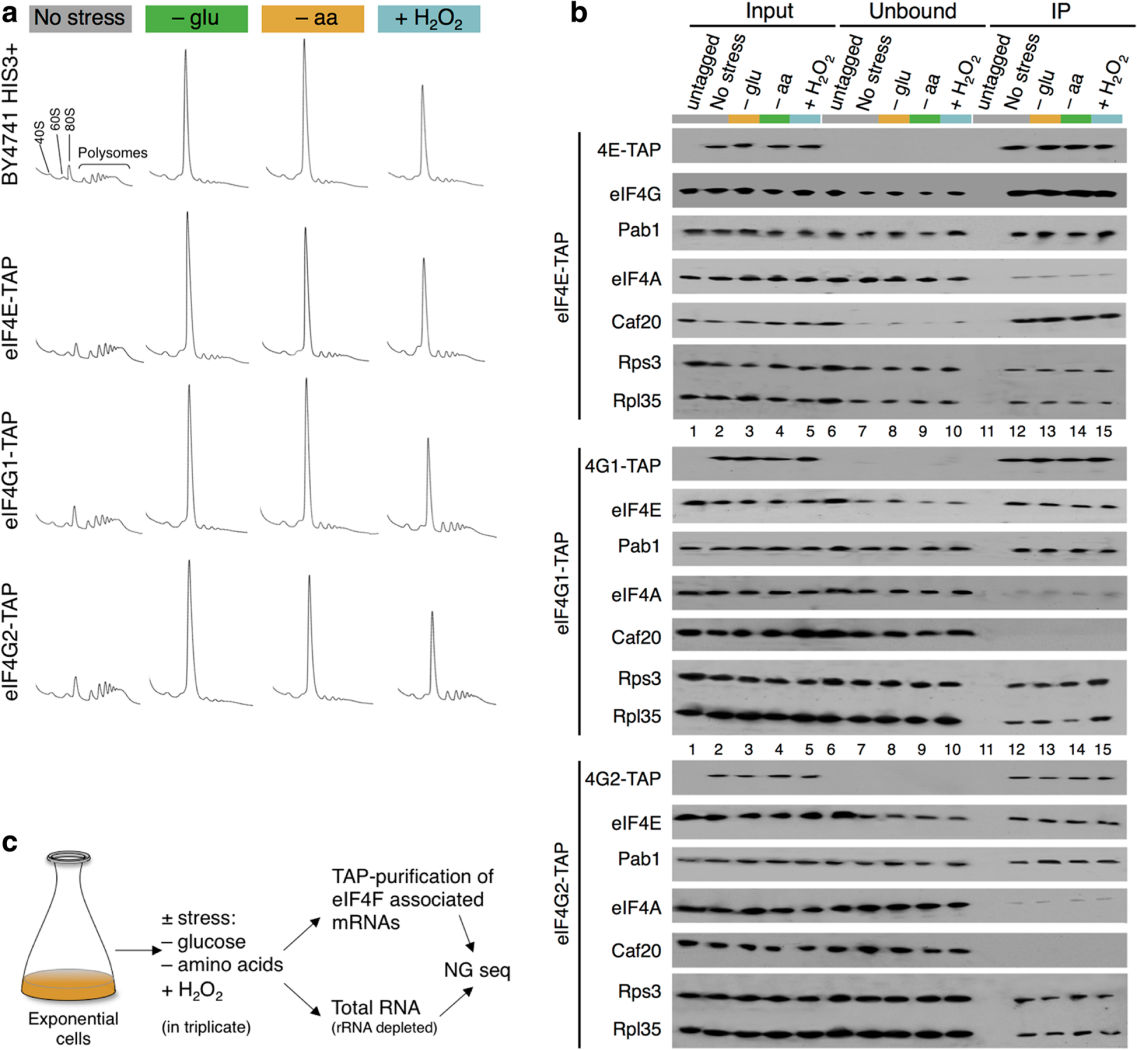
Stress treatment does not affect ‘closed-loop’ factor associations. **a** Polysome profiles of each tagged strain before and following acute stress are unaffected by the TAP tag. **b** TAP-IP recovers equivalent levels of associated factors ± stress. Minor variations seen are not reproducible. **c** Overview of approach to generate samples for RNA sequencing

To assess the effects of stress on eIF4F–mRNA interactions, we selected three stresses that each cause rapid polysome run-off, indicative of translational inhibition at the initiation phase (Fig. 1a, top). Following preliminary experiments, we chose the following experimental set-up. Cells were grown in synthetic complete 2% glucose medium to A_600_ of 0.6, then subjected to one of three different stresses: a 20-min shift to pre-warmed 2% glucose minimal medium lacking all amino acids (–aa); a 10-min shift to synthetic complete medium lacking glucose (–glu); or the addition of 0.4 mM hydrogen peroxide (+H_2_O_2_) for 15 min. These timings were selected to give as similar as possible impact on translation initiation, as judged by polysome profile analyses (Fig. 1a) and to provide consistency with prior studies [26, 27, 30]. As expected all stresses caused an accumulation of inactive 80S monosomes and depletion of ribosomes from the polysomal portion of the sucrose gradients. We used haploid BY4741 strains bearing an individual tandem affinity purification (TAP) tag integrated chromosomally directly downstream of the coding region of eIF4E, eIF4G1 or eIF4G2. TAP tagging did not alter the global impact of stress on polysome profiles (Fig. 1a). We showed previously that these TAP tags do not interfere with factor expression levels or closed-loop factor protein interactions [13]. To extend these analyses we examined protein–protein interactions of each TAP-tagged strain following application of stress. To preserve native interactions, as far as possible, rapid cell harvest and lysis under liquid nitrogen were used. We captured the TAP-tagged protein complexes on IgG-coupled magnetic beads (TAP-IP, see ‘Methods’) and assessed protein–protein interactions with immunoblotting. We were surprised to find that stress apparently changed none of the interactions examined. eIF4E maintained interactions with eIF4G, Pab1, some eIF4A, Caf20 and ribosomal protein markers Rps3/Rpl35 (Fig. 1b). eIF4G1 and eIF4G2 maintained interactions across the stresses applied identically to eIF4E, except for the 4E-BP Caf20, which does not interact with either isoform of eIF4G as expected. Any minor differences between stresses in western signals were not found reproducible across replicates.

### Transcript abundance changes are stress specific and factor independent

We proceeded to examine the mRNAs bound to each factor by affinity capture following stress and next-generation RNA sequencing (RNA-seq). As controls and to monitor stress-induced changes in mRNA abundance, rRNA-depleted total RNA was sequenced (Fig. 1c). All samples were sequenced in triplicate and mapped to the *S. cerevisiae* genome using standard tools (see ‘Methods’). Per gene counts are shown in Additional file 1: Supplementary Source Data 1 and summarised in Additional file 2: Figure S1A. First, we compared total RNA samples to examine changes in transcript abundance following stress. All replicates correlated well with each other; pairwise Pearson correlations between replicates exceeded 0.95 (except eIF4G2 –glu > 0.935). In comparisons between tagged strains, variations are no greater between control unstressed samples and those exposed to the same stress. Only Pearson correlations for –aa samples differ greatly from the other stresses (Additional file 2: Figure S1B). The replicate data were analysed with edgeR to calculate the fold changes in transcript abundance (presented as log_2_ ratios of cpm, here termed ∆T; Additional file 3: Supplementary Source Data 2). Following these short treatment periods, pairwise ∆T plots show that –aa stress has a large impact on the transcriptome (the differences in transcript abundance are spread along a wider range), while –glu and + H_2_O_2_ do not (Additional file 2: Figure S2A). –aa stress targets expected gene categories, significantly downregulating ribosome and protein synthesis genes, while upregulating transport and carbohydrate metabolism in line with previous reports [26, 28]. Hence while each stress has a similar impact on the global polysome profiles, they have different effects on the transcriptomes at these early time points following stress application. As transcription changes more robustly when longer stress periods are used [37, 38], these data indicate that translational repression largely precedes transcriptional reprogramming (Additional file 2: Figure S2B).

### Stress induces changes in relative association of mRNAs with the eIF4F proteins

As outlined in the introduction, our prior analysis of eIF4E and eIF4G1/2 association with mRNAs revealed greater variation between mRNAs than expected [13]. Our stress TAP-IP sequencing samples have Pearson correlations between replicates and across strains subjected to the same stress similar to those for unstressed samples (Additional file 2: Figure S3A). In line with our prior analyses of unstressed TAP-IP samples, edgeR data analyses indicate that there are hundreds of mRNAs significantly differentially enriched or depleted with eIFs 4E, 4G1 and 4G2 compared with total RNA (termed IP/T) following each stress (false discovery rate [FDR] < 0.05; Additional file 2: Figure S3B and Additional file 4: Supplementary Source Data 3).

**Fig. 2:**
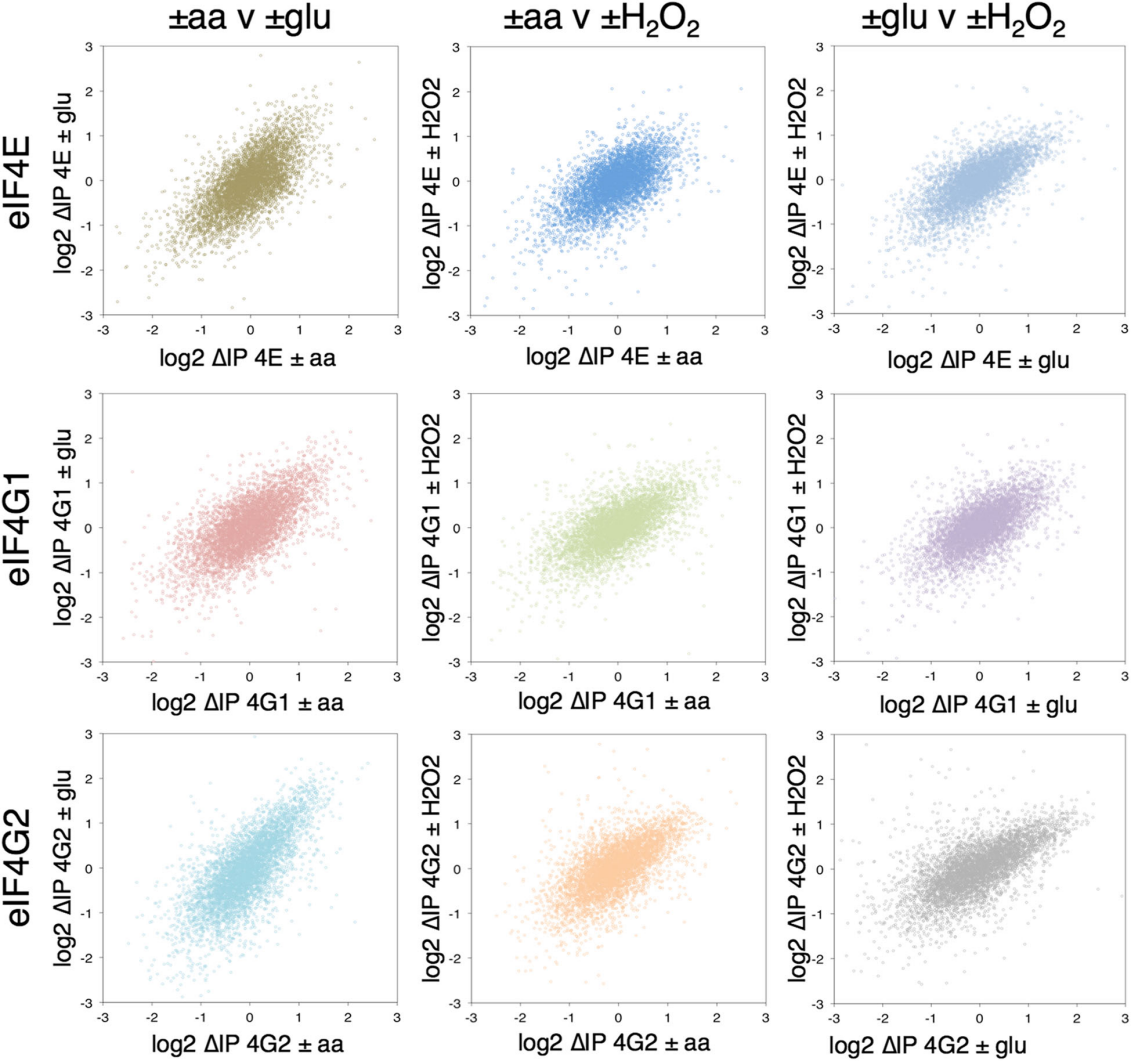
Overall changes in mRNA association with eIF4F following different stresses (∆IP). Pairwise *scatterplots* showing differential association of 5348 mRNAs with each eIF4F factor following stress (log_2_ fold change stress/unstressed: ∆IP). mRNAs where association changes the most following stress are located at the extremes. *Top*: eIF4E plots; *middle*: eIF4G1 plots; *bottom*: eIF4G2 plots. In each row: *left*: ± aa (*x-axis*) plotted against ± glu (*y-axis*); *middle*: ± aa (*x-axis*) against ± H_2_O_2_ (*y-axis*); *right*: ± glu (*x-axis*) against ± H_2_O_2_ (*y-axis*)

To explore changes in eIF4F–mRNA interactions in response to stress we calculated the relative change in each factor’s association in stressed cells vs control unstressed cells. This we term ∆IP (Additional file 5: Supplementary Source Data 4). Figure 2 shows pairwise plots of ∆IP, each as a cloud of 5348 mRNAs, comparing the changes in association of each factor with each stress. The distribution of changes all correlate well (R > 0.6) indicating, for example, that the mRNAs which increase in association with eIF4E –aa also tend to increase in association with –glu, with the same relationship holding for relatively depleted RNAs (Fig. 2, top left). The same trends can be seen across all stress and factor comparisons. These data indicate that relative changes in association of mRNAs with these eIF4F proteins during stress exhibit a common response to environmental stress and the associated rapid translational repression (Fig. 1b). As shown in the plots in Additional file 2: Figure S4B, –glu stress induced the most significant changes, then –aa, with + H_2_O_2_ inducing the fewest changes. This hierarchy of stress effects broadly reflect the degree of impact each stress has on the polysome profile, with + H_2_O_2_ having the weakest impact and –glu having the most (Fig. 1a). This contrasts with the ∆T changes, where –glu and + H_2_O_2_ have modest impact in terms of statistically significant expression changes and only –aa induces a robust transcriptional response (Additional file 2: Figure S4a).

### Anti-correlated changes in transcription (∆T) and eIF4F association (∆IP)

**Fig. 3:**
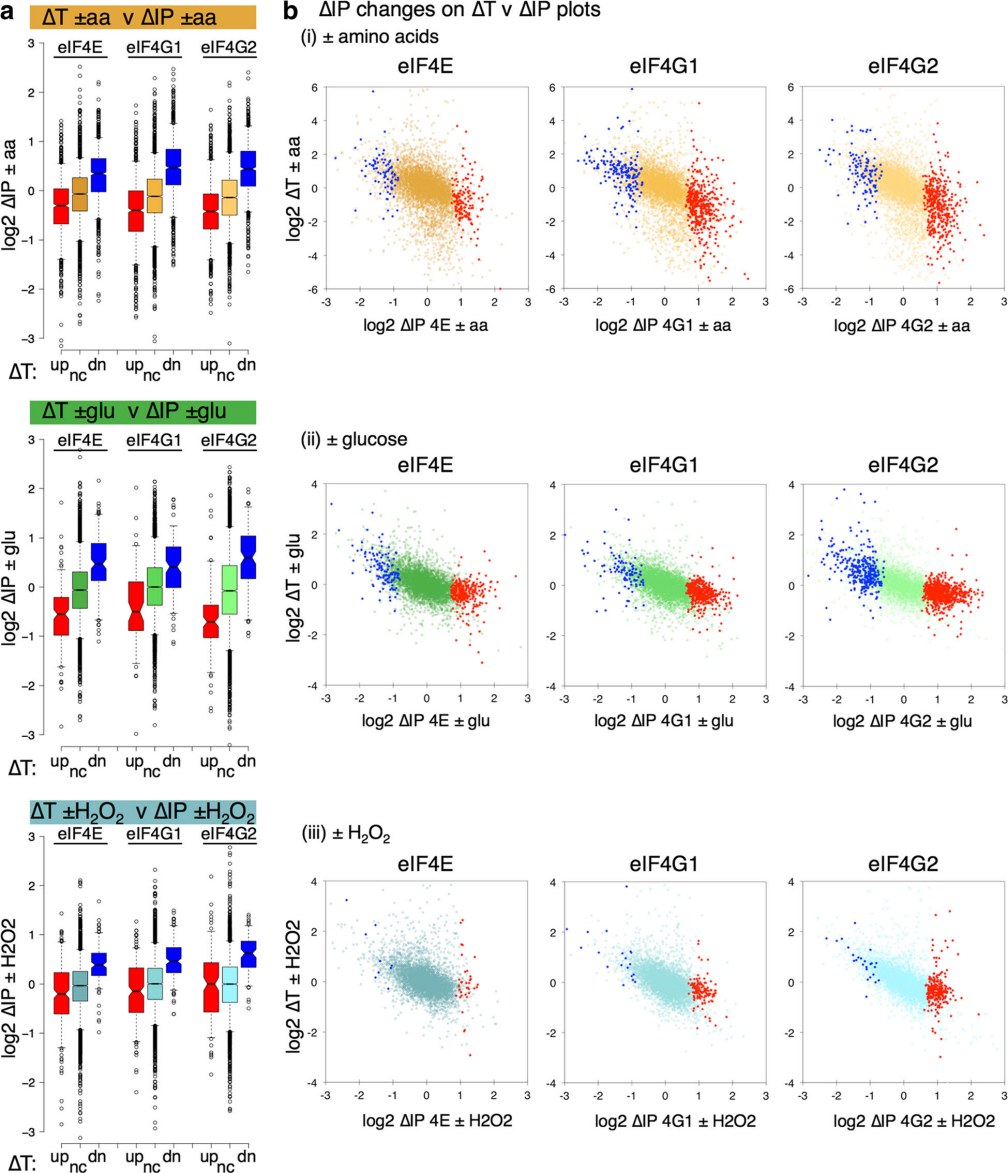
Reciprocal changes in transcription (∆T) and eIF4F association (∆IP) in response to stress. **a** *Box-and-whisker plots* showing the distribution of changes in IP (∆IP) with eIF4F proteins for mRNAs transcriptionally upregulated (up, *red*) downregulated (dn, *blue*) or statistically (FDR < 0.05) not changed (nc, *gold*, *green* or *light blue*) in response to the same stress in the same strain. **b** *Scatter plots* of 5348 mRNAs showing change in transcription (∆T) and change in eIF4F association with stress (∆IP). mRNAs whose factor association changes significantly (FDR < 0.05) following stress are highlighted in *red* (up) and *blue* (down), based on edgeR analyses, see Additional file 5: Supplementary Source Data 4

We compared the stress-induced changes in both transcript levels (∆T) and factor–mRNA associations (∆IP). This revealed an unexpected anti-correlation between ∆T and ∆IP that was shared across the stresses. Figure 3a splits the mRNAs into three classes ∆T up (red), down (blue) and not significantly changed (gold, green or blue) and plots the ∆IP for the genes in each class as a series of box-and-whisker plots. mRNAs depleted following stress (negative ∆T) become relatively enriched with eIF4E, eIF4G1 and eIF4G2 (blue boxes). In contrast, those mRNAs enriched in ∆T are generally depleted for the eIF4F proteins (red boxes). These same trends are seen for all three stresses and this observation still holds when the datasets are split according to significant changes in ∆IP instead of ∆T. Figure 3b shows this as scatterplots. Although there are some mRNA-specific exceptions, the trends are for ∆IP-enriched mRNAs (red points) on the right of each plot to have ∆T < 0, while ∆IP-depleted mRNAs (blue points) have ∆T > 0.

Gene Ontology (GO) comparisons of significantly changing mRNAs in both ∆T and ∆IP using yeast GO Slim categories (Additional file 2: Figure S5) also highlight the opposing natures of ∆T and ∆IP. ∆T changes are largely stress-specific and as expected from previous comparable studies [26, 27, 31]; –aa upregulates carbohydrate metabolism and membrane transport functions and downregulates translation, –glu upregulates carbohydrate transport systems and + H_2_O_2_ induces oxidative stress-response mRNAs. In contrast, ∆IP changes are more typically shared across the stresses and proteins. There are very few GO Slim terms over-represented among the eIF4F-depleted mRNAs (blue cells in Additional file 2: Figure S5, right panel), instead almost all coordinated GO term enrichments appear among those mRNAs whose eIF4F interactions are increased following stress. Thus, we conclude that ∆T and ∆IP changes in eIF4F mRNA association are negatively correlated across the stresses investigated and that decreased eIF4F-mRNA binding following stress is not apparently targeted to specific GO categories.

The current understanding of the role of eIF4F is to promote translation initiation. Frequently, stress changes promote ‘potentiation’ where enhanced translation accompanies and reinforces changes in transcription [39] and this has been observed previously for –aa starvation [26] and glucose depletion [31]. So, it was highly unexpected that the changes in eIF4E and eIF4G association with mRNAs that we observed should oppose the changes in transcript abundance. However, the responses seen are consistent across all three independent stresses applied.

### Anti-correlated changes in ribosome occupancy (∆TE) and eIF4F association (∆IP)

To compare our ∆IP data with changes in translation we were able to use literature-sourced ribosome footprinting datasets for each of our chosen stresses. When sequencing of ribosome-protected RNA fragments was first described as a powerful measure of protein synthesis, Ingolia et al. used the technique to address the role of amino acid starvation [28]. Similarly, Gerashchenko et al. reported the impact of hydrogen peroxide [29] and Zid and O’Shea studied glucose depletion [36]. All experimental data we analysed here employed the same yeast strain and stress protocols were similar, but not identical, to our own (e.g. cells grown at 30 °C and short time-point stresses: –aa 20 min, –glu 15 min, +H_2_O_2_ 30 min). A commonly accepted way to summarise the ribosome footprinting data is to calculate a measure of ribosome engagement with mRNAs by summing the footprints mapped to a gene and dividing by total transcript counts (each normalised to read depth and ORF length). This is widely termed TE. Because bulk polysomes are depleted in stressed cells (Fig. 1), absolute TEs of most RNAs are reduced by stress. However, these effects are dampened by the normalisation of total footprint counts, such that changes in TE or ∆TE calculations are relative rather than absolute. Therefore, genes with a negative ∆TE have a greater than average reduction in translation, while those with a positive ∆TE represent both mRNAs more resistant to repression than average and those mRNAs with enhanced translation under the applied stress.

**Fig. 4:**
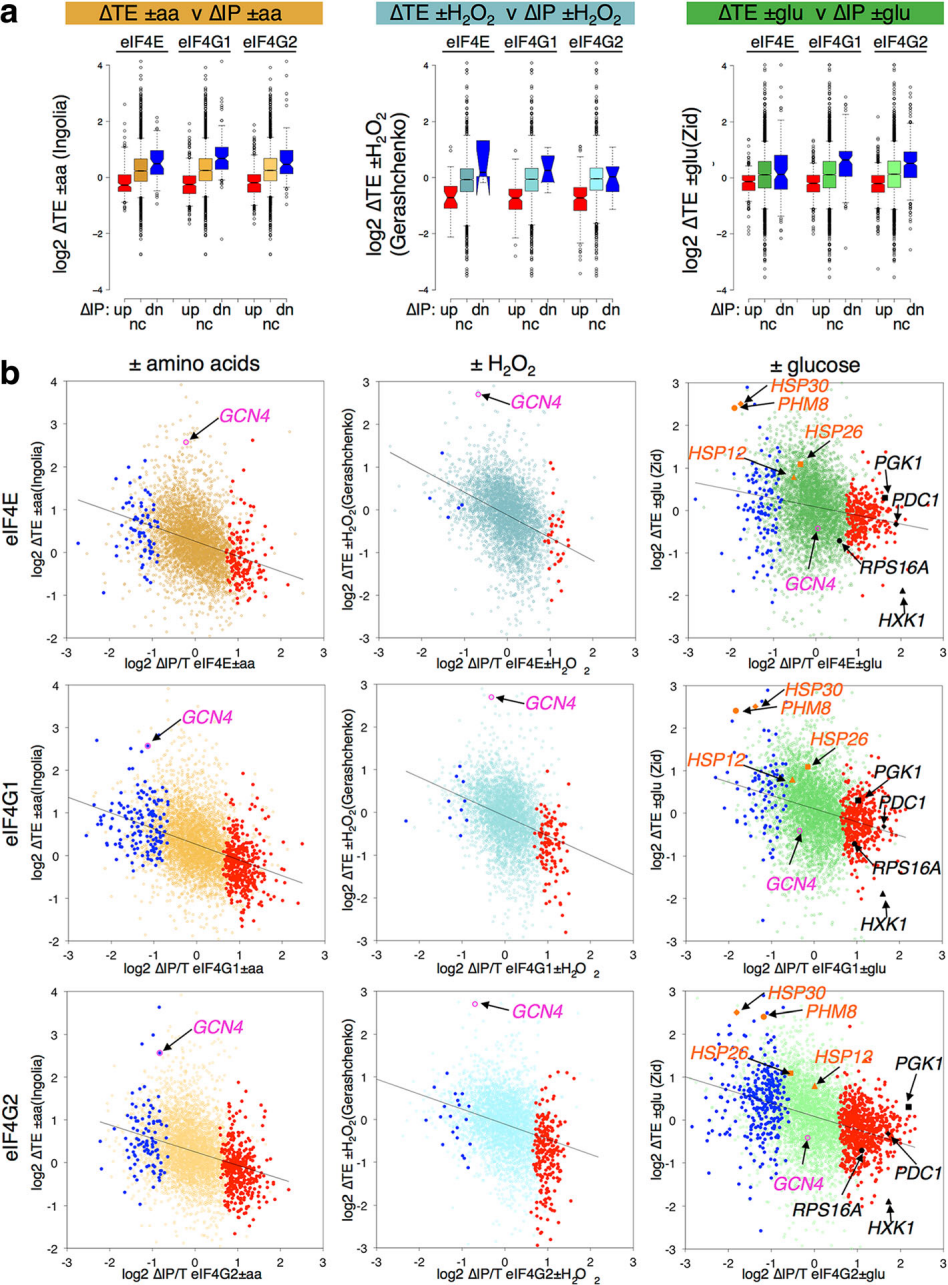
Reciprocal changes in relative ribosome occupancy –translation efficiency (∆TE) and eIF4F association (∆IP) in response to stress. **a** *Box-and-whisker plots* showing the distribution of changes in TE with eIF4F factor association. mRNAs statistically (FDR < 0.05) enriched in IP following stress (up, *red*) depleted (dn, *blue*) or not changed (nc, *gold*, *green* or *blue*) in response to the same stress in the same strain. ∆TE calculated from previously published experiments. **b** *Scatter plots* of the same mRNAs highlighting specific labelled mRNAs discussed in the text

Comparing our ∆IP data with the calculated ∆TE values, we found the same inverse correlations that we observed for comparisons with changes in transcript levels (Fig. 4). This is shown by splitting the ∆IP values into significantly enriched (red, labelled up), significantly depleted (blue, labelled dn) or not significantly changed (gold, green or blue depending on the stress, labelled nc) for all three proteins and plotting the ∆TE values as either box plots (Fig. 4a) or as scatter plots with each mRNA shown (Fig. 4b). While many of the genes that change the most in ∆TE do not change in association with the eIF4F proteins, the trends indicate a clear negative correlation between the ∆TE and ∆IP (black line on each plot). For example, *GCN4* translation is coupled to eIF2 phosphorylation and is enhanced both by –aa and + H_2_O_2_, but not –glu (pink highlighted spot in Fig. 4b plots). *GCN4* mRNA is depleted from both eIF4G1 and eIF4G2 by –aa only (Additional file 5: Supplementary Source Data 4). Therefore, changes in eIF4F–mRNA associations are likely one component of the translational controls that operate during stress responses.

Many mRNAs have been found to accumulate in P bodies and/or stress granules during the glucose starvation response, while others remain cytoplasmically diffuse. Granules formed during stress are believed to be sites where non-translating mRNAs accumulate. For example, *PGK1*, *RPS16A*, *PDC1* and *HXK1* have all been shown to rapidly localise to P bodies following glucose depletion using fluorescence tagging experiments [35, 36]. All these mRNAs become relatively eIF4F-associated in our experiments and tend to have lower ∆TEs (black spots in Fig. 4b, right panels). In contrast, several *HSP* mRNAs remained diffusely cytoplasmic in mRNA localisation studies and maintained high ∆TEs [36]. These mRNAs become relatively depleted of eIF4F proteins, particularly *HSP30* and *PHM8.* Phm8 is a nucleotidase functioning in the ribose salvage pathway that is induced following glucose depletion [31].

Taken together these data indicate that eIF4E and the eIF4G isoforms respond in a coordinated fashion to these different stresses, changing their affinity to a portion of the mRNAs in the cell. The changes in eIF4F binding go against prior expectations, such that those mRNAs enriched with the eIF4F proteins (positive ∆IP) are among those mRNAs that become relatively depleted of ribosomes (negative ∆TE); while those mRNAs that become relatively depleted of eIF4F following stress are among those that are comparatively better engaged with ribosomes. One explanation for the increased stability of eIF4F with translationally repressed mRNAs is the formation of a stable mRNA closed loop enriched in eIF4E and eIF4G on translationally inactive mRNAs. When glucose is depleted these enter P bodies and/or stress granules. In contrast, mRNAs that become relatively depleted in eIF4F proteins are a sub-set of those mRNAs that remain or become better engaged with ribosomes following stress. These mRNAs are reminiscent of a class of mRNA identified in our previous study of closed-loop factor–mRNA interaction in actively growing cells [13]. As outlined in the ‘Introduction’, we previously found a cluster of mRNAs (termed Group I) that were well translated but depleted for the eIF4F proteins. So, to gain further insight into how stress remodels eIF4F–mRNA interactions we examined the fate of the different cluster mRNAs to each stress.

### Group I mRNAs become enriched in eIF4F following stress

**Fig. 5:**
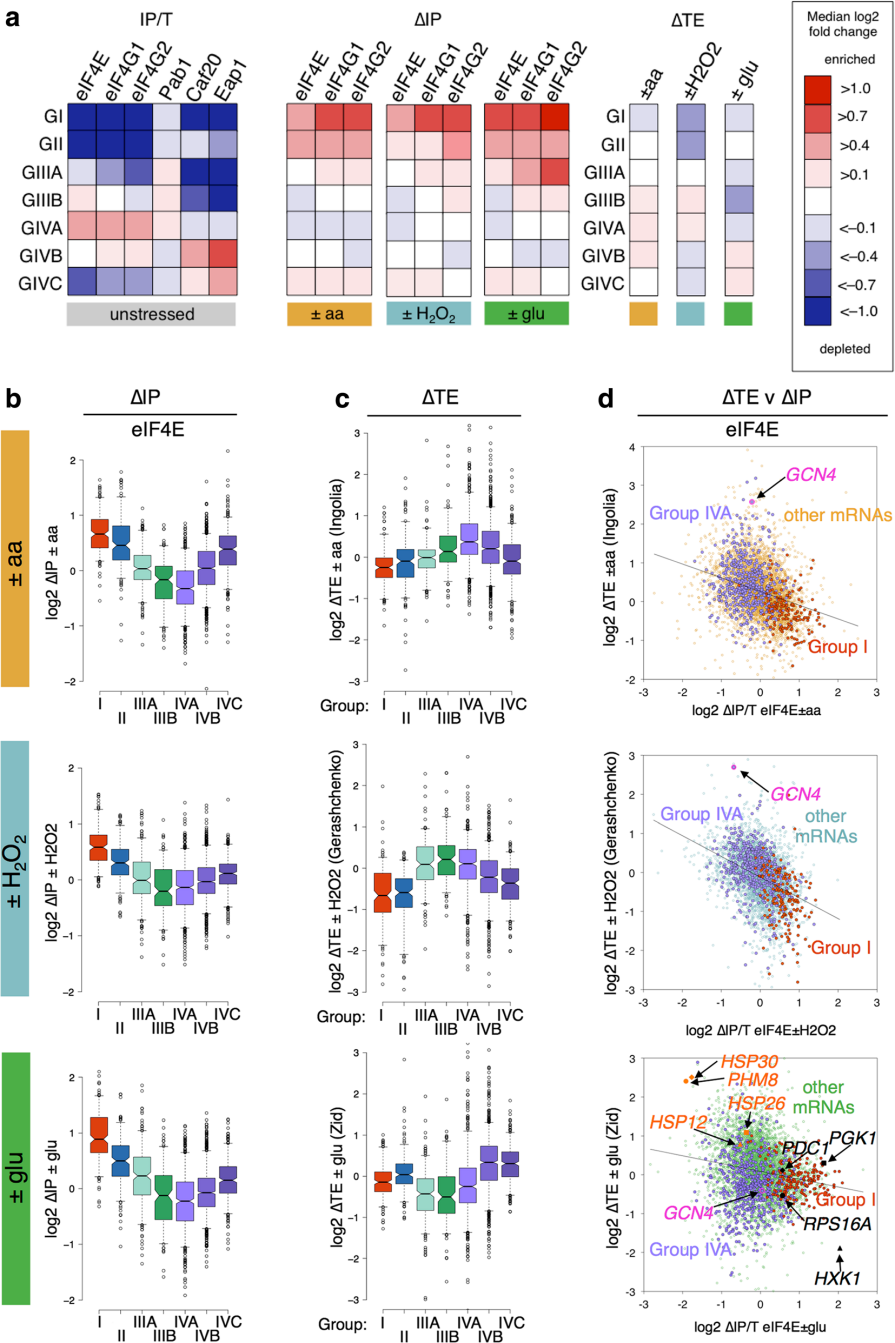
Opposing responses to stress of ‘closed-loop’ Group I and IVA mRNAs. **a** *Left*: median log_2_ fold change for Group I–IVC mRNAs as defined by Costello et al. [13]. See Additional file 2: Figure S6A for box plot representations of this data. ∆IP change in eIF4F association (*middle*) and ∆TE (*right*) are shown for each group across each of the three stresses. Colour key in box. **b**, **c** *Box plots* showing the effect of change in eIF4E association with each stress (**b**) and ∆TE (**c**) on each gene cluster ∆IP denoted by a specific colour (G I *red*; G II *blue*; G IIIA and B shades of *green*; G IVA-C shades of *purple*). **d** *Pairwise plots* showing changes in IP and TE for G I and G IVA for eIF4E. Specific mRNAs are indicated with *arrows*. mRNA groups are denoted by a specific colour (G I *red*; G II *blue*; G IIIA and B shades of *green*; G IVA-C shades of *purple*). **b**–**d** *Top* ± amino acids plots, *middle* ± H_2_O_2_, *bottom* ± glucose. Equivalent plots to panels (**b**) and (**d**) for eIF4G1 and eIF4G2, respectively, are shown in Additional file 2: Figure S6B and C

As outlined in the ‘Introduction’, our previous analyses of closed-loop factor–mRNA complexes identified four broad clusters of mRNAs. The largest clusters Groups III and IV were further subdivided to generate seven cluster groups named I, II, IIIA, IIIB, IVA, IVB and IVC. The distribution of fold changes of mRNAs from different cluster groups when bound to eIF4E, eIF4G1, eIF4G2, Pab1, and the two yeast 4E-BPs Caf20 and Eap1 is shown as a series of box plots in Additional file 2: Figure S6A, with the median fold change for each cluster group summarised in Fig. 5a, left. Additional file 2: Figure S6A shows that eIF4E, eIF4G1 and eIF4G2 are all relatively enriched in groups IIIA and B as well as IVA, B and C, while being relatively depleted in groups I and II. In contrast, the 4E-BPs are particularly enriched in groups IVB and C, as well as to a lesser degree groups II and IVA, but are depleted from groups I, IIIA and B. Despite being well engaged with ribosomes (TE plot in Additional file 2: Figure S6A), Group I RNAs were depleted for eIF4F in unstressed cells.

The impact of stress on factor associations indicates that Group I mRNAs and, to a lesser extent, Group II mRNAs (the clusters most depleted of eIF4F proteins in unstressed cells) become relatively enriched with eIF4E in response to all three stresses (Fig. 5b, red and blue boxes). Equivalent trends are observed for eIF4G1 and 4G2 (Fig. 5a and Additional file 2: Figure S6B). At the same time, these mRNA groups become relatively translationally repressed according to ∆TE measurements (Fig. 5a and c). This suggests that the reciprocal effects seen in Fig. 4 on ∆TE and ∆IP associated with translational repression following multiple stresses are linked with changes in eIF4F associations with mRNAs in Group I, and to a lesser extent Group II. In contrast, Groups IIIB, IVA and IVB that were more stably associated with eIF4F in unstressed IPs tend to be the most depleted groups following stress; though the degree of change for these groups is typically quite modest. At least some of these changes are stress-specific. For example, when the scatterplots shown in Fig. 4 are re-plotted to highlight cluster Group I and Group IVA mRNAs, it is clear that there are reciprocal changes in both eIF4F factor associations and ∆TE for –aa and + H_2_O_2_ (red and lilac spots in Fig. 5d and Additional file 2: Figure S6C). Group IVA changes in ∆TE are more varied in the –glu ∆TE data from Zid and O’Shea [36] (bottom panels). This may be because –glu does not induce translation initiation repression by eIF2 phosphorylation, unlike the other two stresses examined [27, 30]. Finally, Group III mRNAs (especially IIIB) were originally classified as relatively enriched in eIF4F with high TE and were later shown to have short ORFs (Additional file 2: Figure S6A). Group III mRNAs appear largely resistant to change in IP (Fig. 5b) and only have low ∆TE following –glu (Fig. 5c). Therefore, each of the previously identified closed-loop group mRNAs not only have altered factor associations in unstressed cells, they also each exhibit different responses to stress.

### Anti-correlation between eIF4F association and calculated initiation times

**Fig. 6:**
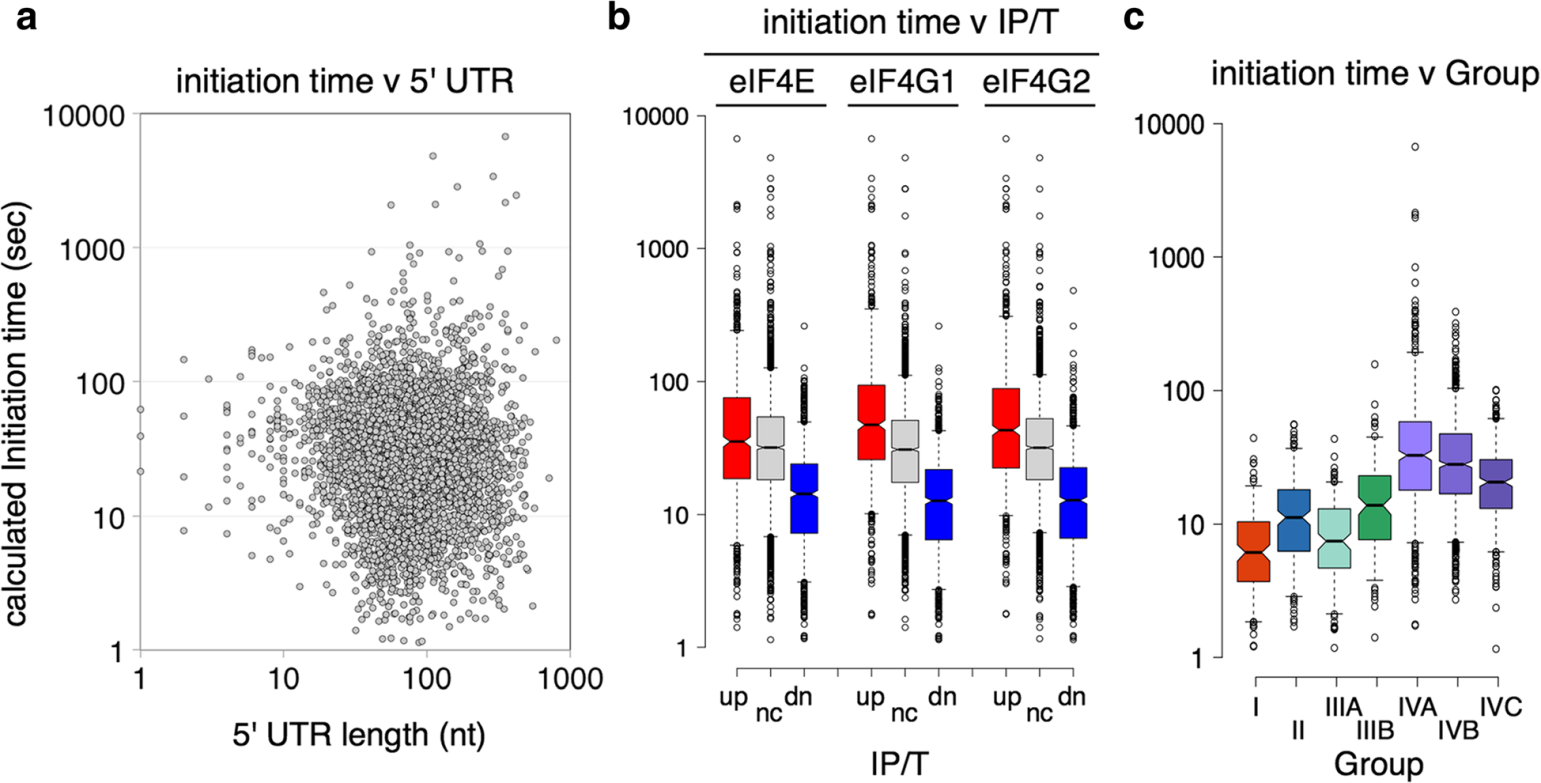
Calculated translation initiation times anti-correlate with eIF4F association in unstressed cells. **a** No correlation between length of 5′ UTR and initiation time calculated from ribosome profiling experiments in unstressed cells [12]. **b** Anti-correlation between eIF4F factor association and calculated initiation time. **c** Mean calculated initiation time across Costello et al. [13] mRNA Groups I–IVC

Our data suggest that some mRNAs change their associations with eIF4E and eIF4G in a coordinated way in response to stress that appears to anti-correlate with expectations based on the known roles of eIF4E and eIF4G in promoting protein synthesis. In the general initiation model, mRNAs bound by eIF4E and either eIF4G1 or eIF4G2 are able to recruit 43S ribosomes to the 5′ cap. Then scanning is initiated during which the 5′ UTR is traversed and an AUG initiation codon is located. At AUG recognition, a reorganization of the initiation complex and release of translation factors occurs to facilitate 60S joining [4]. To connect our eIF4F–mRNA associations with rates of initiation on individual mRNAs, we compared our unstressed data to computed initiation times generated for individual mRNAs in actively growing unstressed cells [12]. Siwiak and Zielenkiewicz developed a quantitative model for translation elongation speed for individual ribosomes on over 4000 mRNAs and used it in combination with ribosome footprinting experimental data to infer the mean time required for each initiation event, to account for both the experimentally observed ribosome density and calculated elongation speed on each mRNA. The calculated initiation times were typically in the range of approximately 5–130 s, with some outliers calculated as taking significantly longer. We plotted these calculated initiation times against 5′ UTR lengths. This generated a wide scatter with no clear correlation, suggesting that scanning time was not likely the most rate-limiting factor for initiation time in this model (Fig. 6a). It is known that yeast mRNAs tend to have short and generally unstructured 5′ UTRs [4]. In contrast, a clear anti-correlation was observed between calculated initiation times and eIF4F factor associations, such that mRNAs depleted for eIF4F have the fastest predicted initiation rates (Fig. 6b). Among the clustered group mRNAs, Groups I and IIIIA have the fastest predicted initiation speeds and Group IVA the slowest (Fig. 6c). As the model only accounts for translation in exponentially growing cells, we could not extend it to our stressed experimental data. Nevertheless, the comparisons agree with our analyses of the stressed factor associations and suggest that, with some exceptions, eIF4F–mRNA associations appear generally less stable on actively initiating mRNAs.

## Discussion

Our experiments have addressed how interactions between mRNAs and the eIF4F proteins (eIF4E and the two eIF4G forms) are altered in response to three acute stresses that each cause a global translation initiation inhibition, specifically nutrient withdrawal (–glu and –aa) or response to a toxic oxidant (+H_2_O_2_) (Fig. 1). We used a relatively simple metric of the degree of mRNA association with TAP-tagged proteins captured on magnetic IgG coupled beads. Many mRNAs change in their associations with the eIF4F proteins (Fig. 2). In accord with prior studies there appears to be a large overlap and broad agreement between the mRNAs binding each eIF4G isoform [13, 40]. While there may be some gene-specific differences between the eIF4G isoform interactions with some mRNAs, the global changes in RNA–protein association that we observe are generally shared between the factors and stresses, suggesting that the eIF4E–eIF4G complex is responding globally to stress and/or translation inhibition as a single unit rather than as individual proteins (Fig. 2). This common global response to environmental stress that changes eIF4F–mRNA interactions likely precedes the well-known common transcriptional response to multiple environmental stresses described previously [37, 38]. This is because at the short times following stress examined here (10–20 min), cells have not yet induced fully their transcriptional response programs.

When we compared a range of mRNA features across the ∆IP significantly enriched (*P* < 0.05) and depleted gene sets, including 5′ UTR and 3′ UTR lengths, we reported secondary structure and for enrichments for known RNA-binding proteins. No clear enrichments were found (not shown). This is perhaps unexpected, but likely reflects that eIF4E binds primarily to the 5′ cap structure in yeast and that eIF4G-binding to eIF4E enhances this cap affinity (K_D_ = < 15–20 nM) [8, 41, 42] to promote 40S ribosome recruitment and protein synthesis initiation. Many studies show that eIF4E and eIF4G interaction with mRNA promote its translation. It was therefore against expectations that the changes in factor–mRNA associations induced in response to stress are anti-correlated with both transcription (Fig. 3) and translation changes (Fig. 4). By these measures of gene expression, it appears that mRNAs which become more associated with both eIF4E and eIF4G are among those mRNAs that are translationally repressed by stress. Our data specifically point to classes of mRNA that are normally well translated, but depleted of eIF4F in unstressed cells, that become relatively more enriched in eIF4F and relatively poorly translated in stressed cells (Group I and to a lesser extent Group II mRNAs; Fig. 5). At the same time mRNAs that become less associated with eIF4F are among those mRNAs relatively better translated in response to stress (Figs. 4 and 5). Calculated overall initiation times are also lower for mRNAs depleted for eIF4F in unstressed cells (Fig. 6). Hence, these results appear to run counter to widespread prior observations that eIF4E and eIF4G function to promote translation initiation [1, 8, 43].

In thinking of how to explain these observations, it is important to consider both eIF4E-dependent and -independent mechanisms that may account for our observed eIF4F depletion on specific mRNAs and the reciprocal changes induced by stress and translation inhibition. eIF4E-independent mechanisms include the use of alternative cap-binding proteins. For example, it has recently been found that human eIF4E has differential mRNA specificity dependent upon the 5′ terminal nucleotide of the mRNA [44]. A test RNA beginning with a C residue had lower affinity for eIF4E than RNAs beginning with A or G. In addition, transcripts beginning with C had lower ribosome occupancy [44]. However, C-initiating transcripts, including 5′ TOP mRNAs, can bind preferentially to the 5′ cap-C–binding DM15 domain within human LARP1 [45]. These studies indicate that specific mRNAs may interact with cap-binding proteins other than eIF4E. However, such a mechanism appears unlikely to explain our findings, because (1) both yeast LARP proteins, Slf1 and Sro9, lack DM15 related regions [46]; and (2) the defined mRNA groups I–IVC show no altered preferences in mapped 5′ end nucleotides (analysis not shown). In yeast, other known 5′ cap interacting proteins include the predominantly nuclear cap-binding complex (CBC) of Cbc1/Sto1 and Cbc2, equivalent to mammalian Cbp80 and Cbp20, respectively. The CBC is involved in mRNA splicing, transcription termination, mRNA export and the pioneer round of translation [47]. In addition, deletion of *CBC1* was shown to enhance translational repression and delay the cellular response to hyperosmotic stress [48], although there is no report of a specific role for the CBC in any of the stresses studied here. Second, the Dcp1 and Dcp2 decapping complex that removes the 5′ cap structure from mRNAs during mRNA decay also binds the cap before its cleavage [49, 50]. *PGK1* is a Group I mRNA and is one of the key relatively stable mRNAs upon which the general mRNA-decay model is derived. This model indicates that poly A tail shortening precedes decapping and 5′ - 3′ exonucleolytic decay [49, 50]. It follows therefore that deadenylated mRNAs should have lower Pab1-binding, but we found Pab1 binding enhanced on Group I mRNAs (Additional file 2: Figure S6A; [13]). So, as a whole, our data are not compatible with current understanding of known alternative cap-interacting proteins explaining Group I mRNA behaviour, so they are not discussed further.

It was demonstrated recently that the d subunit of eIF3 possesses 5′ cap binding activity and it was further shown that eIF3-led 43S recruitment promotes c-Jun mRNA translation [51]. Thus, a similar means could provide an alternative explanation for the behaviour of Group I mRNAs. However, yeast eIF3 does not have an eIF3d orthologue [4] and so a directly analogous mechanism appears unlikely. However, because yeast eIF3 possesses RNA-binding domains [4] and can enhance recruitment of 40S ribosomes to mRNAs independently of the eIF4F complex [8, 20], eIF3-promoted mRNA recruitment remains one possible explanation for low eIF4F retention on Group I mRNAs. How yeast eIF3 could protect the 5′ cap to resist RNA decay remains unresolved.

If not initiation via an eIF4E-independent route, then how could an eIF4F-dependent mechanism account for our findings? There is evidence that there are multiple forms of closed-loop complex and that some states are more readily disrupted than others (see below). Canonical translation initiation typically has several main steps: (1) eIF4F–mRNA interactions; (2) recruitment of 43S complexes; (3) scanning; (4) AUG codon recognition; and (5) 60S subunit joining. Only one initiation event is thought to occur on an individual mRNA at any one time, so highly translating mRNAs likely cycle rapidly between successive rounds of initiation.

**Fig. 7:**
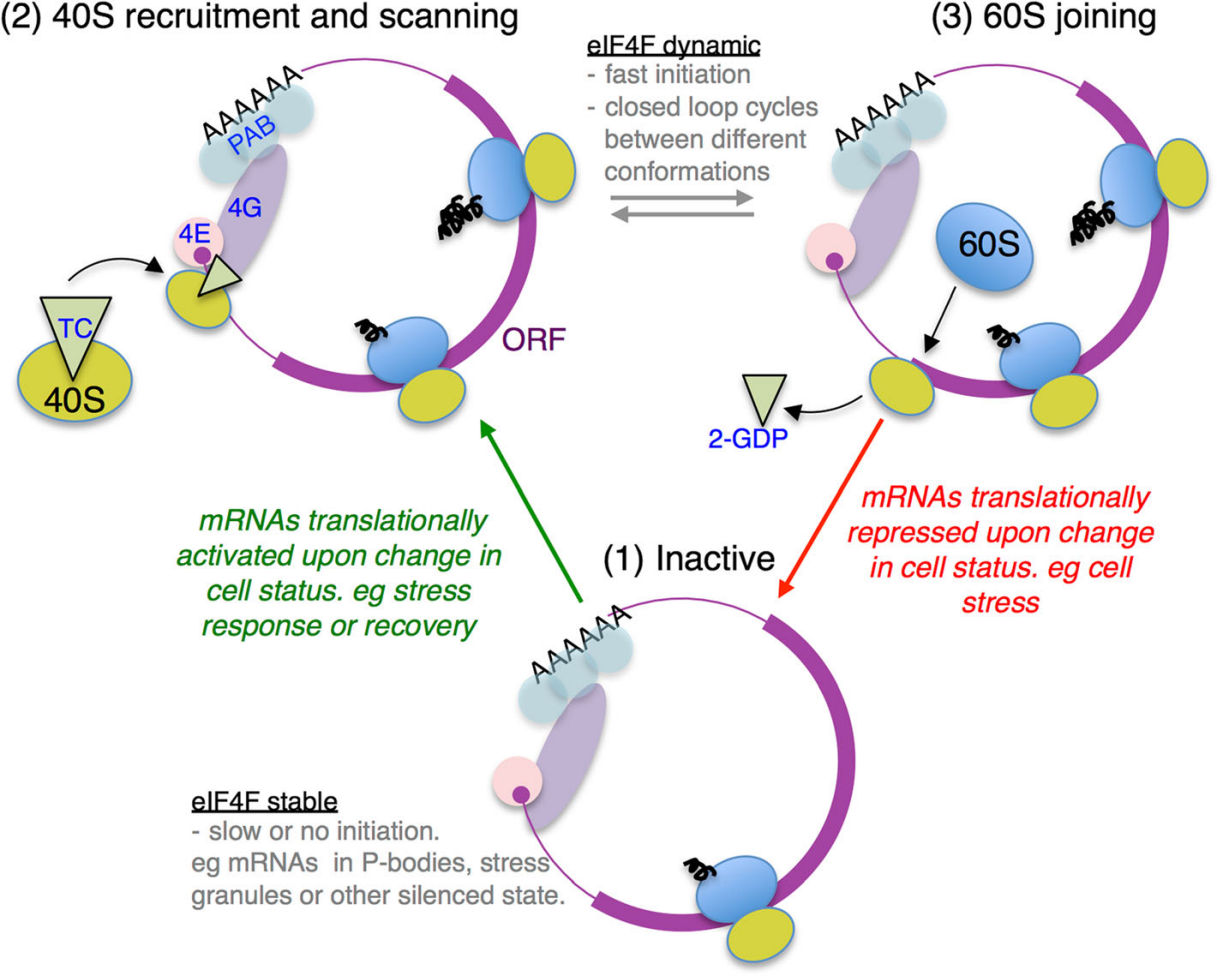
Model for differential eIF4F associations during translation initiation. *Diagram* depicts three different states of closed-loop mRNA complex. *Bottom*: State 1: a non-initiating mRNA. *Top left:* State 2: a 48S bound complex with contacts between eIF4G and recruited 43S factors during which scanning occurs. *Top right:* State 3: a post-AUG recognition complex undergoing 60S joining where initiation factors including eIF2-GDP are released. Actively initiating mRNAs likely cycle between the conformations 2 and 3 (*grey arrows*), correlating with low eIF4F affinity or recovery. mRNAs initiating rapidly under optimal growth conditions, but sensitive to stress transition to a conformation where eIF4F becomes more stably associated and TE lowered: *red arrow* to state 1. Other mRNAs have high affinity for 4 F and are initiated less frequently (state 1), but can be activated to recruit more ribosomes following stress, entering a dynamic state where 4 F affinity is lowered: *green arrow* to state 2

It is energetically unfavourable for eIF4F to disassemble and reform for each successive initiation cycle. As eIF4G binding to eIF4E enhances its affinity for mRNA promoting a stable RNA–protein complex [41, 42], it was proposed that this complex may function to promote multiple rounds of ribosome loading [41]. eIF4E/eIF4G binding mRNAs as a unit is also consistent with our prior genome-wide analyses of factor–mRNA interactions, where both factors were co-enriched or depleted from individual mRNAs isolated from unstressed cells [13]. Therefore, in the absence of other stabilising factors, there are likely three main eIF4F–mRNA complex states: (1) non-initiating, or awaiting 40S recruitment; (2) after 40S recruitment during scanning and AUG recognition; and (3) post-AUG recognition during 60S joining (see Fig. 7). As eIF4G makes contacts with the recruited 43S pre-initiation complex, 43S interactions will likely alter the stability of eIF4F–mRNA complexes. Similarly, on 60S joining to form 80S complexes, 40S–eIF4F contact is likely lost. We envision that the most actively initiating mRNAs will transition rapidly between these states (2 and 3). Post-initiating mRNAs where 40S recruitment is slow will also spend time in state 1 awaiting new 40S recruitment. Translationally inactive mRNAs, for example those relocating to P bodies and/or stress granules following glucose starvation, could be variants of state 1 [33–35] as there is widespread ribosome run-off following stress (Fig. 1).

Previous, mainly in vitro, studies support the idea of distinct eIF4F–mRNA complexes during the initiation cycle. In vitro translation reactions in yeast identified distinct 48S and 80S forms of closed-loop complexes containing eIF4E, eIF4G and Pab1 [52]. In addition, evidence for a distinct 60S joining ‘closed-loop’ complex was found using reporter RNAs electroporated into cells containing various mutated translation factors [53]. Similarly, rabbit reticulocyte extract translation of 5′ capped poly A^–^ RNA recently showed that eIF4F was bound to 48S complexes in sucrose gradients, but released from 80S complexes [54]. These data support the idea that the closed-loop complex is remodelled upon 60S subunit joining. Mechanistically, this may entail disruption of 40S-eIF4G interactions that would promote both 60S joining and free eIF4G to recruit a new 40S complex. To explain our data, we propose that one of these states is less stable to capture in our RIP-seq experiments. Thus, rapidly initiating mRNAs become relatively depleted in eIF4F in our experimental system, which therefore provides a convenient tool to identify these RNAs (Fig. 6b).

In this model, in unstressed cells, Group I mRNAs are actively translated and cycle between closed-loop states 2 and 3 (Fig. 7). During acute stress, these mRNAs become translationally repressed and spend more time in state 1 (red arrow in Fig. 7), which is stable to capture in our assay. Likewise, there are other RNAs such as those in Group IVA that are relatively poorly translated in unstressed actively growing cells, but relatively enhanced in eIF4F interactions. In our model, these RNAs are spending longer in state 1 in unstressed cells, but have relatively enhanced translation during stress and lower eIF4F association (Fig. 6c and Additional file 2: Figure S6C). Hence these mRNAs become activated from state 1 into state 2–3 during stress (green arrow in Fig. 7). Presumably recovery from stress and resumed translation and cell growth would reverse these changes. This is different to a previous proposal for differential enrichment of eIF4F/Pab1 with various mRNAs where the mRNA closed loop was suggested to be transient and only prevalent primarily during mRNA activation and becoming less widespread during steady state translation [55].

Importantly, not all mRNAs fit this model. Many mRNAs appear resistant to reciprocal changes in TE and eIF4F IP. Most notable are those in RNA classes IIIA and IIIB. These short mRNAs are relatively stably bound to eIF4F and are well translated in unstressed cells. As indicated in the introduction, these mRNAs are enriched in ribosomes bearing the 40S associated factor Asc1/RACK1 suggesting that Asc1/RACK1 helps direct ribosomes to bind these mRNAs to promote their translation [17]. As eIF4F interactions for Group IIIA and B mRNAs appear relatively unaffected by stress (Fig. 6), it is possible that Asc1/RACK1 acts to maintain the closed loop on these mRNAs. As Asc1 has been shown to recruit other RNA-binding proteins to ribosomes, such as Scp160 [56], further networks of RNA–protein interactions will likely contribute to the modulation of eIF4F–mRNA interactions and translational responses to stress.

## Conclusion

We show that 5′ cap-associated eIF4F proteins change mRNA interactions as part of a coordinated common early translational response to environmental stresses. The eIF4F–mRNA interactions are dynamic and, unexpectedly, generally oppose changes in relative translation and transcription. The data are compatible with a model where multiple mRNA-eIF4F complexes can form with differing stability. Hence, in the absence of other eIF4F stabilizing factors, rapid translation initiation on mRNAs correlates with less stable eIF4F interactions, while the converse is seen for repressed mRNAs.

## Methods

### Strains and growth conditions

eIF4G1 and eIF4G2 TAP-tagged His^+^ strains in the BY4741 background were obtained from Open Biosystems. An untagged *HIS3* BY4741 control strain (GP6001) was used as a control for all TAP experiments [57]. Because the eIF4E-TAP strain altered eIF4E levels a new strain was made using standard cre-lox procedures in GP6001 creating an eIF4E-TAP-tagged strain (GP6312) retaining its native 3′ UTR and a lox scar, as described previously [13]. Strains were grown at 30 °C in synthetic complete dextrose media lacking histidine [SCD–his] grown to A_600_ = ~0.6. Stress treatments included harvesting cells by rapid centrifugation in a warm centrifuge and resuspending in pre-warmed SD minimal medium (–aa, for 20 min) or SC–his medium lacking glucose (–glu for 10 min) or by addition of 0.4 mM H_2_O_2_ (+H_2_O_2_ for 15 min) before cell harvest.

### Polysomal profiling

*S. cerevisiae* was grown to an OD_600_ = 0.6 and, where appropriate, cells were stressed as described above. Cycloheximide was added to a final concentration of 0.1 mg/mL immediately before cell harvest by centrifugation. Cells were lysed into polyribosomal buffer containing cycloheximide and 5 OD_260_ units were loaded onto a 15–50% sucrose gradient, poured as previously described [58].

### TAP-affinity purifications

TAP-affinity purifications were performed as described previously [13, 57]. Briefly, yeast cultures were grown to A_600_ = ~0.6, pelleted, snap frozen in liquid nitrogen and ground in Buffer A (20 mM Tris-HCl [pH 8], 140 mM NaCl, 1 mM MgCl_2_, 0.5% NP40, 0.5 mM DTT, 1 mM PMSF, EDTA free Protease Inhibitor cocktail tablet (Roche), 100 μM NaV_3_O_4_, 5 mM NaF and 40 units/mL RNAsin) using liquid nitrogen and a 6870 Freezer Mill (Spex). Lysates were cleared through two centrifugation steps (15,000 x *g* at 4 °C) and quantified using Bradford Reagent. Ten milligrams of mg total protein were loaded onto Rabbit IgG coupled Tosyl-activated Dynabeads M-280 magnetic beads (Dynal) to ensure maximum depletion of the tagged protein from each extract. Coupling of Rabbit IgG to Tosyl-activated Dynabeads M-280 magnetic beads and TAP affinity purification was performed as previously described [13]. After the final wash, the beads were re-suspended in 270 μL Buffer A. A 20-μL aliquot of the sample was set aside for western blot analysis, and RNA was purified from the remaining 250 μL for RNA-seq.

### Western blot analyses

Protein samples were mixed with 2 x SDS loading dye and heated to 95 °C for 10 min to dissociate protein complexes from the IgG Tosyl-activated Dynabeads M-280 magnetic beads. IP samples were resolved by SDS–PAGE, electroblotted onto nitrocellulose membrane and probed using the relevant primary antibody. TAP tagged proteins were detected using an HRP-conjugated primary antibody to Protein A (Abcam, Cambridge, MA, USA). All other primary antibodies were detected with horseradish peroxidase (HRP)-conjugated rabbit secondary antibody, except Pab1, which was detected using HRP-conjugated mouse secondary antibody as previously described [13].

### RNA sequencing

Triplicate samples of TAP-associated RNA were isolated on M-280 magnetic beads as described above. For RNA target identification, after the final wash, the beads were resuspended in 270 μL Buffer A. A 20-μL aliquot of the sample was set aside for western blot analysis and RNA was purified from the remaining 250 μL. Total (T) RNA and paired immune precipitated (IP) RNA were isolated using a standard Trizol Reagent (Life Technologies, Carlsbad, CA, USA) protocol and re-suspended in 10 μL diethylpyrocarbonate (DEPC) treated water. RNA was quantified using a Nanodrop 8000 spectrophotometer (Thermo Fisher Scientific, Waltham, MA, USA). rRNA was then depleted from both total and IP RNA samples using the Ribominus™ Eukaryote Kit for RNA-seq (Life Technologies, Carlsbad, CA, USA). Depleted samples were ethanol precipitated, washed twice with 70% ethanol and resuspended in 10 μL DEPC water. rRNA depletion was assessed on a 2100 Bioanalyzer (Agilent Technologies, Palo Alto, CA, USA) using a RNA nanochip and the remaining RNA stored at –80 °C. Sequencing libraries were generated using the whole Transcriptome Library Preparation protocol provided with the SOLiD® Total RNA-Seq Kit (Life Technologies, Carlsbad, CA, USA) as described by the manufacturer. DNA libraries were deposited on slides and sequenced using the SOLiD v4 sequencing system (Life Technologies, Carlsbad, CA, USA) at the University of Manchester FBMH Genomic technologies core research facility or at BGI Genomics (Shenzhen, China).

Sequencing reads were mapped to the *S. cerevisiae* genome (genome assembly EF4 downloaded from ENSEMBL) using Bowtie [59] and counts for each transcript were calculated using HTseq [60]. Counts were imported into edgeR where we used the Generalised Linear Model (GLM) in order to test for statistical differences between stresses [61]. We performed three different statistical analyses: (1) ∆T represents differences in transcript abundance between stressed (s) and unstressed (u) samples (T_s_/T_u;_); (2) IP/T represent target-identification experiments, which were performed comparing the abundance of pull-down mRNAs with that of the total transcripts (IP_s_/T_s_ and IP_u_/T_u_); and (3) ∆IP represents changes in target binding following stress (IP_s_/T_s_/IP_u_/T_u_). Unstressed IP_u_/T_u_ results were reported previously [13]. Statistical tests involving corresponding IP and T samples were performed using a paired design. All gene lists were generated using FDR < 0.05 as a significance threshold for inclusion. Sequencing data of stress experiments have been deposited at ArrayExpress (E-MTAB-5836) [62]. Data from unstressed experiments are deposited in ArrayExpress (E-MTAB-2464) [63].

### Data analyses

GO analyses of RNA gene lists from edgeR files were done using the web-based GeneCodis tools (http://genecodis.cnb.csic.es/) to assess the yeast GO Slim functional enrichments of eIF4F associated mRNAs against a reference list of 5348 common yeast ORFs [64, 65]. All reported *P* values were corrected for multiple testing using the Benjamini–Hochberg FDR method. A FDR cut off of 0.05 was used.

Published ribosome profiling data [28, 29, 36] were obtained from the appropriate database deposited files and processed to determine TE, defined as the normalised mean number of ribosome protected fragments mapped to an ORF, divided by the number of transcripts per million reads for that ORF, and ∆TE defined as the change in TE in stress conditions divided by TE under unstressed conditions from the same datasets. The –aa and + H_2_O_2_ datasets were from duplicate experiments, while only a single –glu replicate was available as other samples in this study used either a different yeast strain or poly A selection rather than RNA-depletion. Poly A selection has been shown to have sequencing bias with yeast samples [66] so cannot be readily compared with our rRNA depleted data.

Initiation times calculated [12] for 4531 mRNAs were compared with unstressed IP/T data for eIF4E and eIF4G. Box-and-whisker plots were drawn using the BoxPlotR online tool (http://shiny.chemgrid.org/boxplotr/). Centre lines show the medians; box limits indicate the 25th and 75th percentiles as determined by R software; whiskers extend to 5th and 95th percentiles, outliers are represented by dots. The notches are defined as ± 1.58 × interquartile range/square root (n). This represents the 95% confidence interval for each median. Non-overlapping notches give roughly 95% confidence that two medians differ.

## Additional files

Additional file 1: Supplementary Source Data 1. Counts by gene after analysis of mapped counts by HTSEQ. Transcript counts of mapped reads for each replicate of each experiment. (XLSX 2793 kb)

Additional file 2: Figures S1–S6. Supplementary Figures. Figures and legends for Supplementary Figures S1–S6. (PDF 7144 kb)

Additional file 3: Supplementary Source Data 2. ∆T edgeR files for changes in transcript levels with stress. Each TAP-tagged strain ± each stress is shown in a different tab. edgeR outputs shown for Total stress/Total control (∆T). (XLSX 4301 kb)

Additional file 4: Supplementary Source Data 3. IP/T edgeR files for enrichment of RNAs in TAP IP with stress. Each TAP-tagged strain IP/T for stressed and unstressed samples is shown in a different tab. edgeR outputs shown for IP/Total (IP/T). (XLSX 5767 kb)

Additional file 5: Supplementary Source Data 4. ∆IP edgeR files for changes in IP with stress. Each TAP-tagged strain ± each stress is shown in a different tab. edgeR outputs shown for [IP(stress)/Total(stress)]/[IP(control)/Total(control)] (termed ∆IP). (XLSX 4435 kb)
