# Supplementary material for: Dynamic changes in eIF4F-mRNA interactions revealed by global analyses of environmental stress responses
Source: Genome Biol. 2017 Oct 27;18:201. doi: 10.1186/s13059-017-1338-4 (PMC5660459; doi:10.1186/s13059-017-1338-4)

Additional File 2: Figures S1-S6.

Dynamic changes in eIF4F–mRNA interactions revealed by global analyses of environmental stress responses

Joseph L. Costello, Christopher J. Kershaw, Lydia M. Castelli, David Talavera, William Rowe, Paul F. G. Sims, Mark P. Ashe, Christopher M. Grant, Simon J. Hubbard, and Graham D. Pavitt

Legends to Supplementary Figures

Additional file 2: Figure S1. Summary of HTseq mapped counts per mRNA and Pearson correlations between total RNA samples sequenced.

A. Millions of counts mapped to each total (T) and TAP-tagged protein associated (IP) RNA-seq sample. Triplicate biological repeats (1-3) per tagged factor eIFs 4E, 4G1 and 4G2.

B. Unclustered chart of Pearson correlation of counts per million between all total RNAseq samples. Shows excellent agreement between replicates and across the three TAP strains. Only –aa stress samples differ greatly from other stresses and unstressed samples.

Additional file 2: Figure S2: Only –aa starvation shows widespread transcript changes to stress.

A. Pairwise plots of log mean fold change (stressed/unstressed) from edgeR processed RNAseq data for 5348 common mRNAs across three biological repeats. Shows similarity of responses across the TAP strains –aa stress impacts most on transcript abundance at the time point sampled. **B**. Summary of number of mRNAs significantly changing abundance following stress from edgeR analysis of 5348 common mRNAs across three biological repeats. FDR <0.01 and 0.05 shown. edgeR files in Source Data File 2.

Additional file 2: Figure S3. Pearson Correlations between IP RNAseq samples.

A. Pearson correlation of cpm between all IP RNAseq samples, similar to the Supplementary Figure 1B comparison between total samples. **B**. Numbers of significantly enriched and depleted (*FDR* <0.05) total mRNAs (left, ΔT) and mRNAs associated with each TAP-tagged factor. See Source Data files 2 and 3 for full analyses.

Additional file 2: Figure S4. mRNAs that significantly change in association with eIF4F following stress.

A. Number of mRNAs that change significantly FDR <0.05 in association with eIF4F proteins following stress (ΔIP). **B**. Scatter plots of 5348 mRNAs $\log_2$ IP/T unstressed v stress for the three cap-binding

factor IPs. mRNAs with association changes significantly(FDR <0.05) following stress are highlighted in red (increase) and blue (decrease).

Additional file 2: Figure S5. GO Slim comparison of changes in transcription and eIF4F factor association.

Analyses comparing GO Slim enrichments of ΔT (left) and ΔIP (right) enriched and depleted mRNAs, performed as described in Methods.

Additional file 2: Figure S6. Changes in TE and IP responses of 'closed loop' associated gene groups I and IV to stress.

A. Box plots showing IP/T for 2767 mRNAs split into seven cluster groups (Groups I-IVC) defined in Figure 4 from Costello *et al* [10], with differential enrichment with the closed-loop proteins eIF4E, eIF4G1, eIF4G2, Pab1, Caf20 and Eap1. Plots shown here are based on edgeR fold change calculations (see unstressed samples in Source Data File 3), while the original clustering analysis [10], was performed on individual replicate IP and T paired samples. TE [58] and ORF length plots for the seven clusters also shown (bottom). Box plots are colored as in Figure 5. **B**. Box-plots showing the effect of change in eIF4G1 and eIF4G2 association with each stress on each gene cluster ΔIP. Each group is colored as in Figure 5. **C**. ΔIP v TE plots showing Group I and IVA mRNAs for eIF4G1 and eIF4G2.

Figure S1

A

	4E 1		4E 2		4E 3		4G1 1		4G1 2		4G1 3		4G2 1		4G2 2		4G2 3	
	T	IP	T	IP	T	IP	T	IP	T	IP	T	IP	T	IP	T	IP	T	IP
unstressed	9.1	12.0	6.8	5.9	1.5	2.8	18.8	16.4	4.8	4.4	9.8	12.1	7.8	7.2	3.5	12.4	6.6	27.8
– glu	7.1	10.6	13.6	11.4	1.2	9.8	10.3	29.9	5.9	6.8	12.2	29.2	7.8	13.9	4.8	15.1	2.8	7.6
– aa	1.2	2.6	7.5	19.3	1.3	2.5	6.7	19.6	3.6	11.8	9.1	16.9	10.2	7.0	5.8	20.4	3.9	16.5
+ H2O2	2.2	4.5	9.9	14.3	1.5	1.6	5.5	14.5	12.7	11.8	8.8	22.1	1.9	9.6	5.1	23.4	9.5	20.7

B

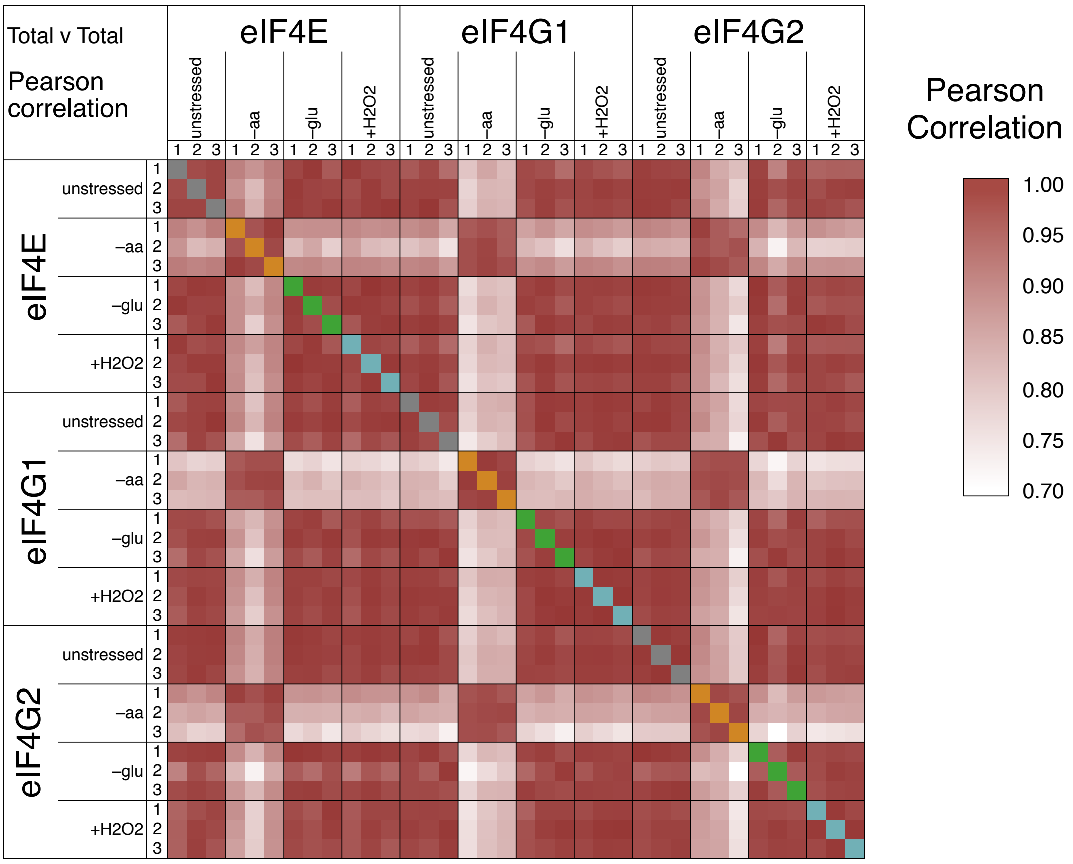

Figure S2

A

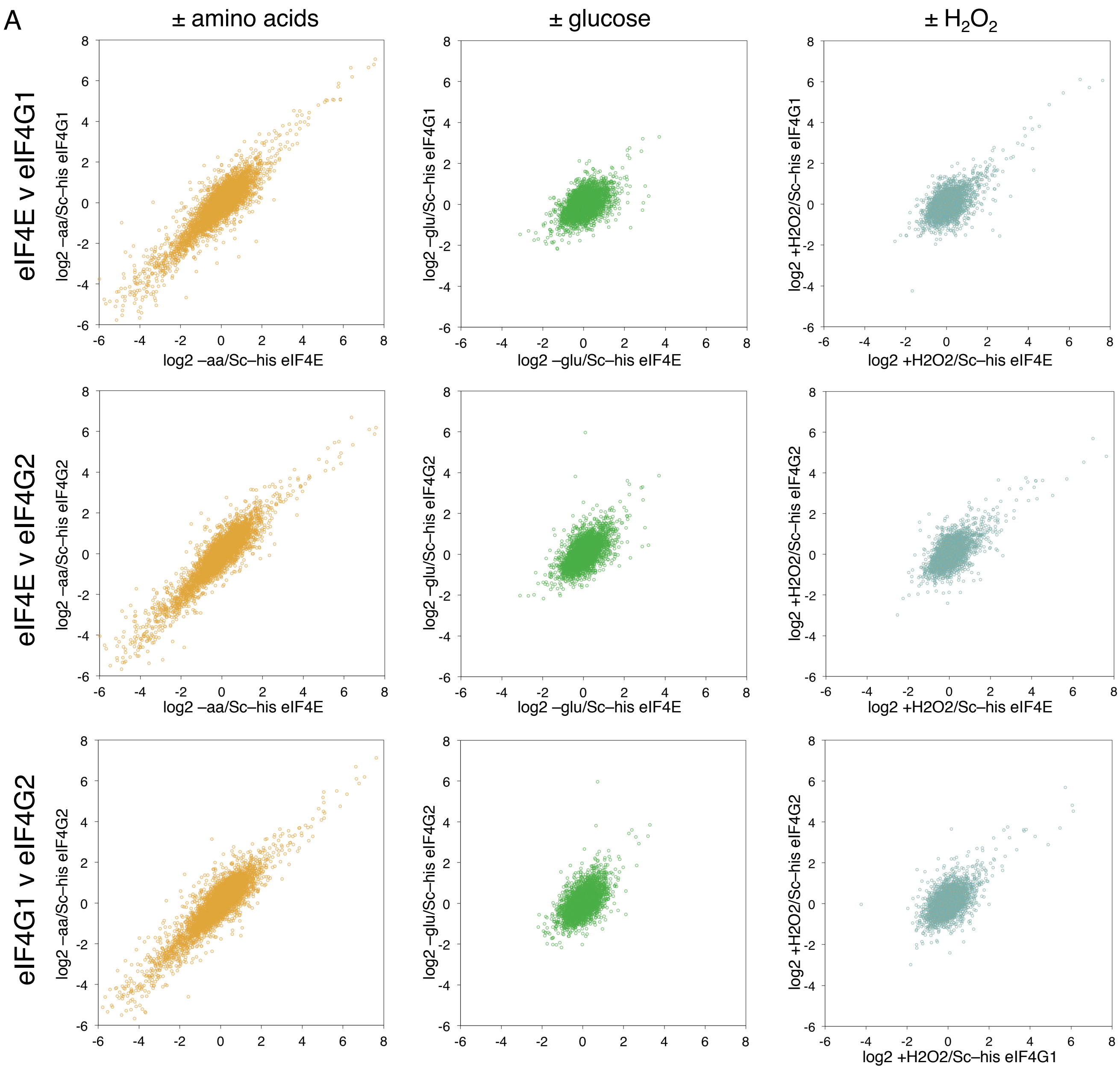

B

ΔT log2 –aa/Sc–his eIF	– aa			– glu			+ H2O2		
	4E	4G1	4G2	4E	4G1	4G2	4E	4G1	4G2
Up FDR<0.01	683	666	604	85	40	78	182	143	87
Up FDR<0.05	999	953	899	154	70	117	240	210	138
Down FDR<0.05	1124	1245	1141	155	121	113	145	175	108
Down *FDR* <0.01	929	1056	963	86	66	46	76	82	43

Figure S3

A

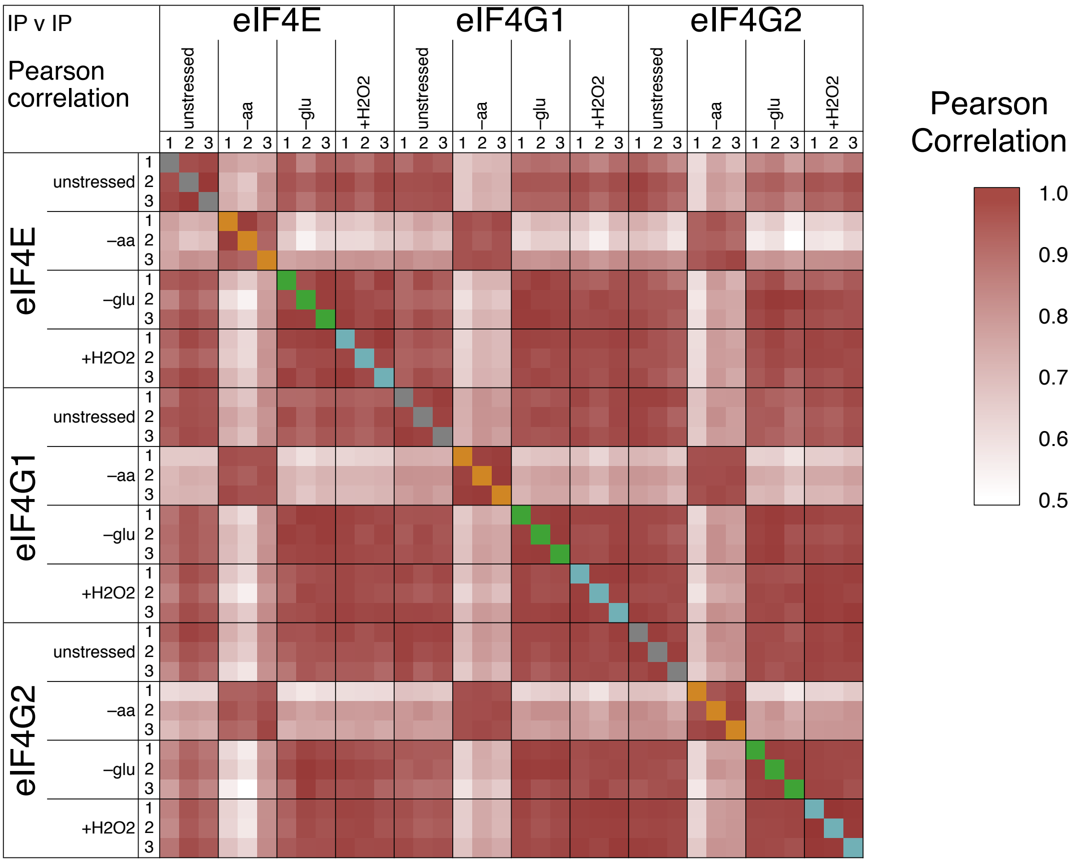

IP v IP
Pearson correlation
eIF4E
eIF4G1
eIF4G2
unstressed
–aa
–glu
+H2O2
1 2 3
Pearson Correlation
1.0
0.9
0.8
0.7
0.6
0.5

B

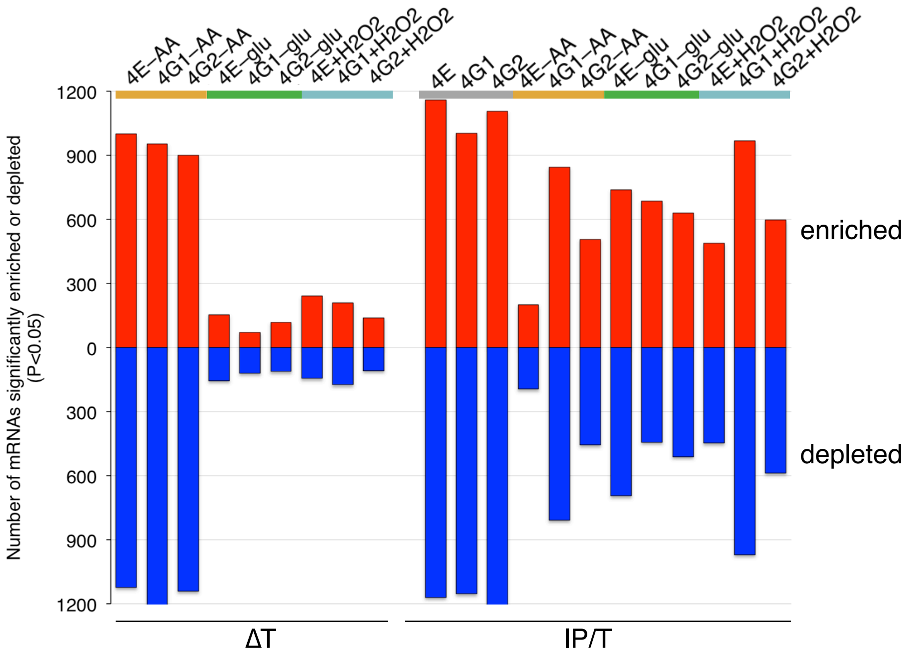

4E–AA
4G1–AA
4G2–AA
4E–glu
4G1–glu
4G2–glu
4E+H2O2
4G1+H2O2
4G2+H2O2
4E
4G1
4G2
4E–AA
4G1–AA
4G2–AA
4E–glu
4G1–glu
4G2–glu
4E+H2O2
4G1+H2O2
4G2+H2O2
Number of mRNAs significantly enriched or depleted (P<0.05)
1200
900
600
300
0
300
600
900
1200
enriched
depleted
ΔT
IP/T

Figure S4

A

ΔIP FDR	± amino acids 4E	4G1	4G2	± glucose 4E	4G1	4G2	± H2O2 4E	4G1	4G2
Up <0.05	150	428	403	303	400	708	36	127	259
Down<0.05	80	178	120	124	103	311	9	19	25

B

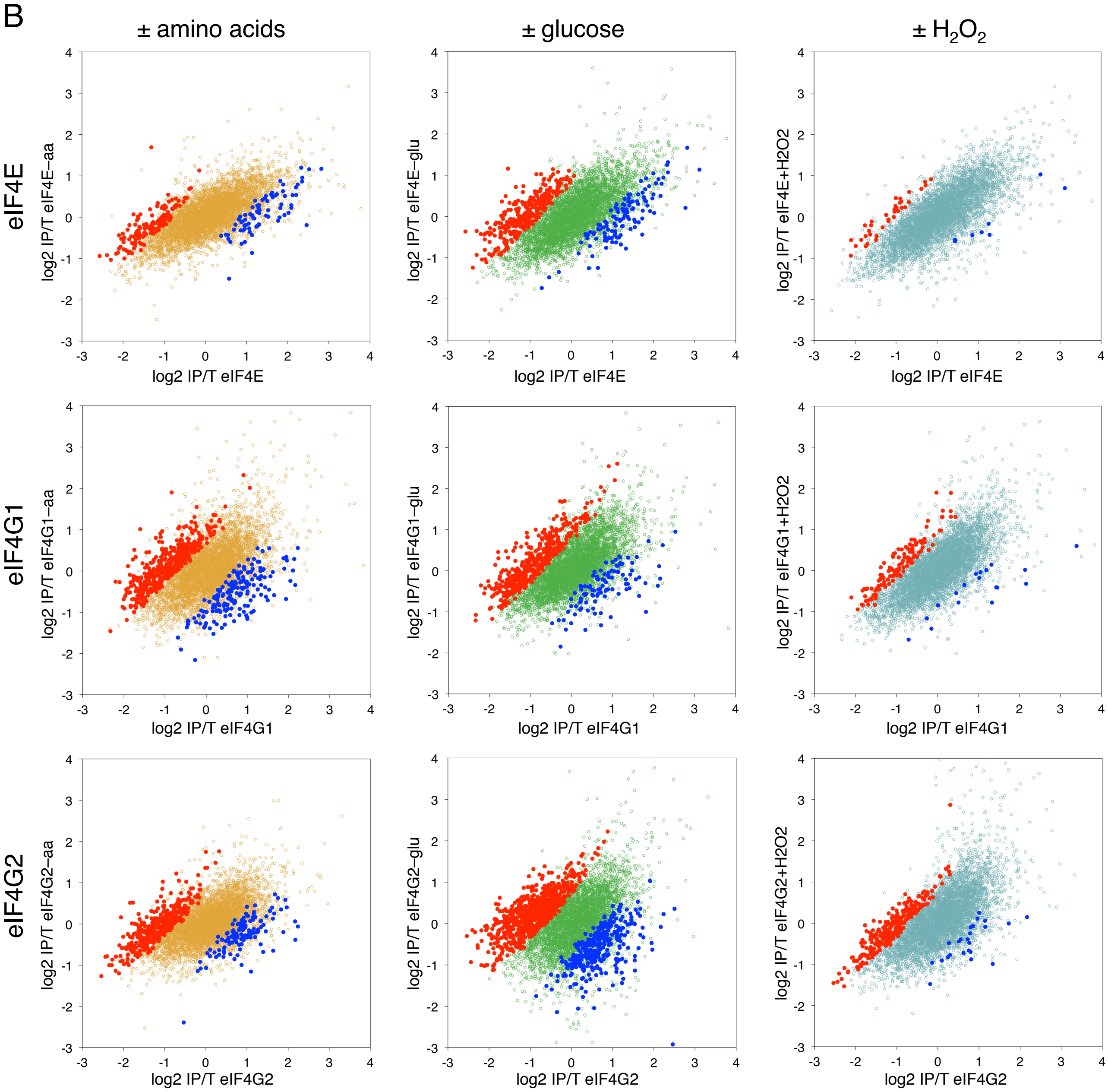

Figure S5

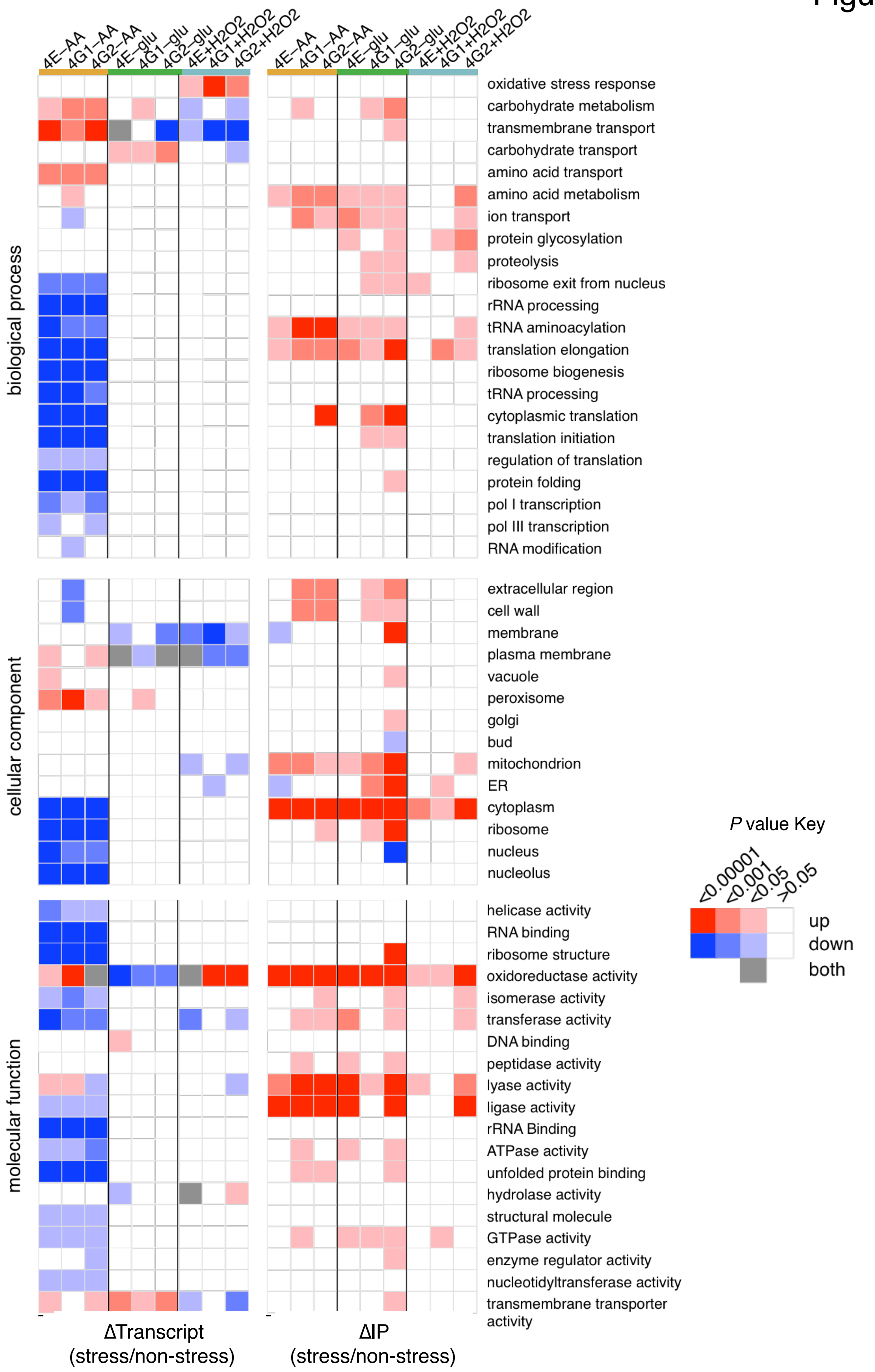
4E–AA
4G1–AA
4G2–AA
4E–glu
4G1–glu
4G2–glu
4E+H2O2
4G1+H2O2
4G2+H2O2
biological process
oxidative stress response
carbohydrate metabolism
transmembrane transport
carbohydrate transport
amino acid transport
amino acid metabolism
ion transport
protein glycosylation
proteolysis
ribosome exit from nucleus
rRNA processing
tRNA aminoacylation
translation elongation
ribosome biogenesis
tRNA processing
cytoplasmic translation
translation initiation
regulation of translation
protein folding
pol I transcription
pol III transcription
RNA modification
cellular component
extracellular region
cell wall
membrane
plasma membrane
vacuole
peroxisome
golgi
bud
mitochondrion
ER
cytoplasm
ribosome
nucleus
nucleolus
molecular function
helicase activity
RNA binding
ribosome structure
oxidoreductase activity
isomerase activity
transferase activity
DNA binding
peptidase activity
lyase activity
ligase activity
rRNA Binding
ATPase activity
unfolded protein binding
hydrolase activity
structural molecule
GTPase activity
enzyme regulator activity
nucleotidyltransferase activity
transmembrane transporter activity
ΔTranscript (stress/non-stress)
ΔIP (stress/non-stress)
P value Key
<0.00001
<0.001
<0.05
>0.05
up
down
both

Figure S6A

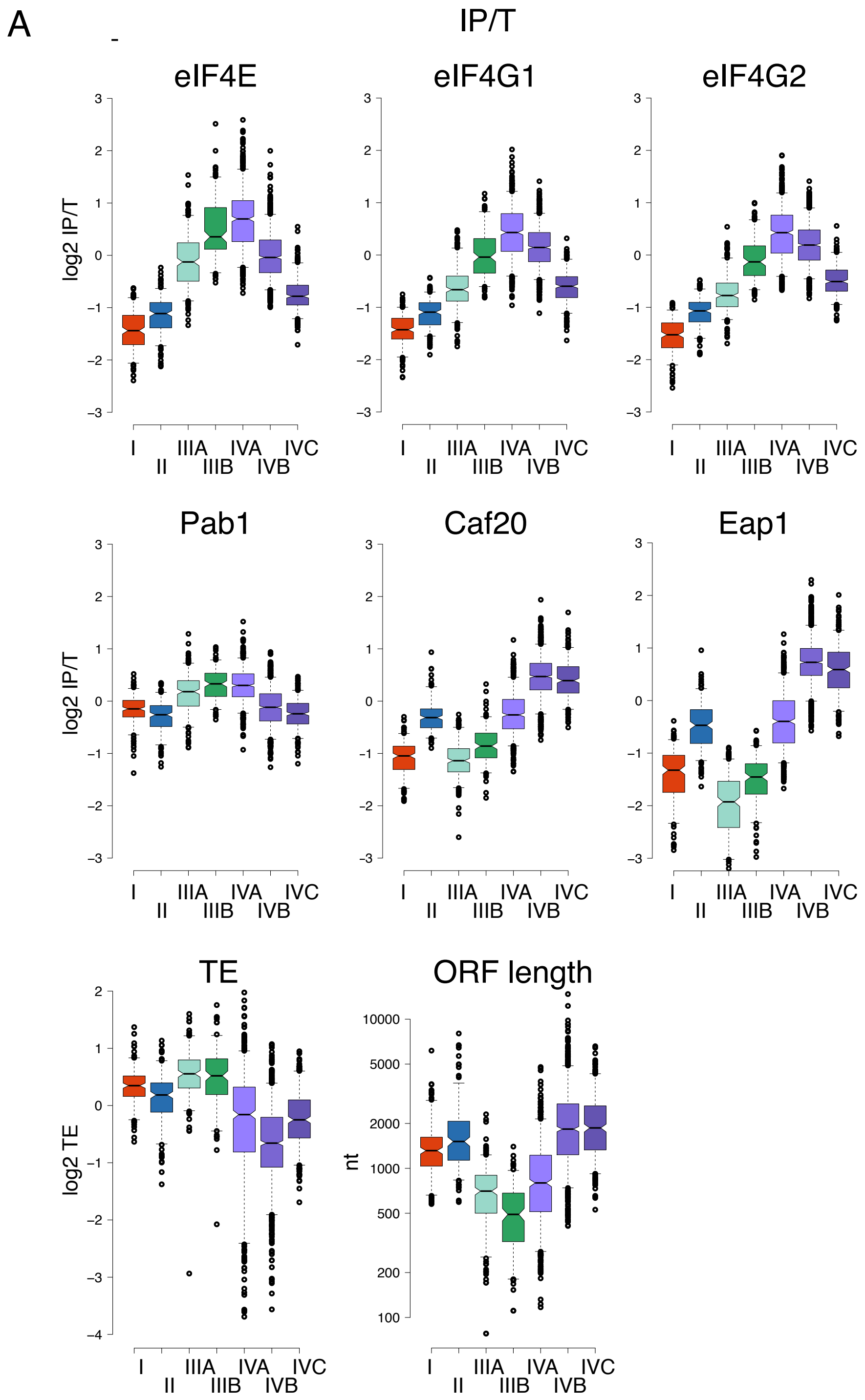

A
IP/T
eIF4E
eIF4G1
eIF4G2
log2 IP/T
Pab1
Caf20
Eap1
log2 IP/T
TE
log2 TE
ORF length
nt
I
II
IIIA
IIIB
IVA
IVB
IVC

Figure S6B and S6C

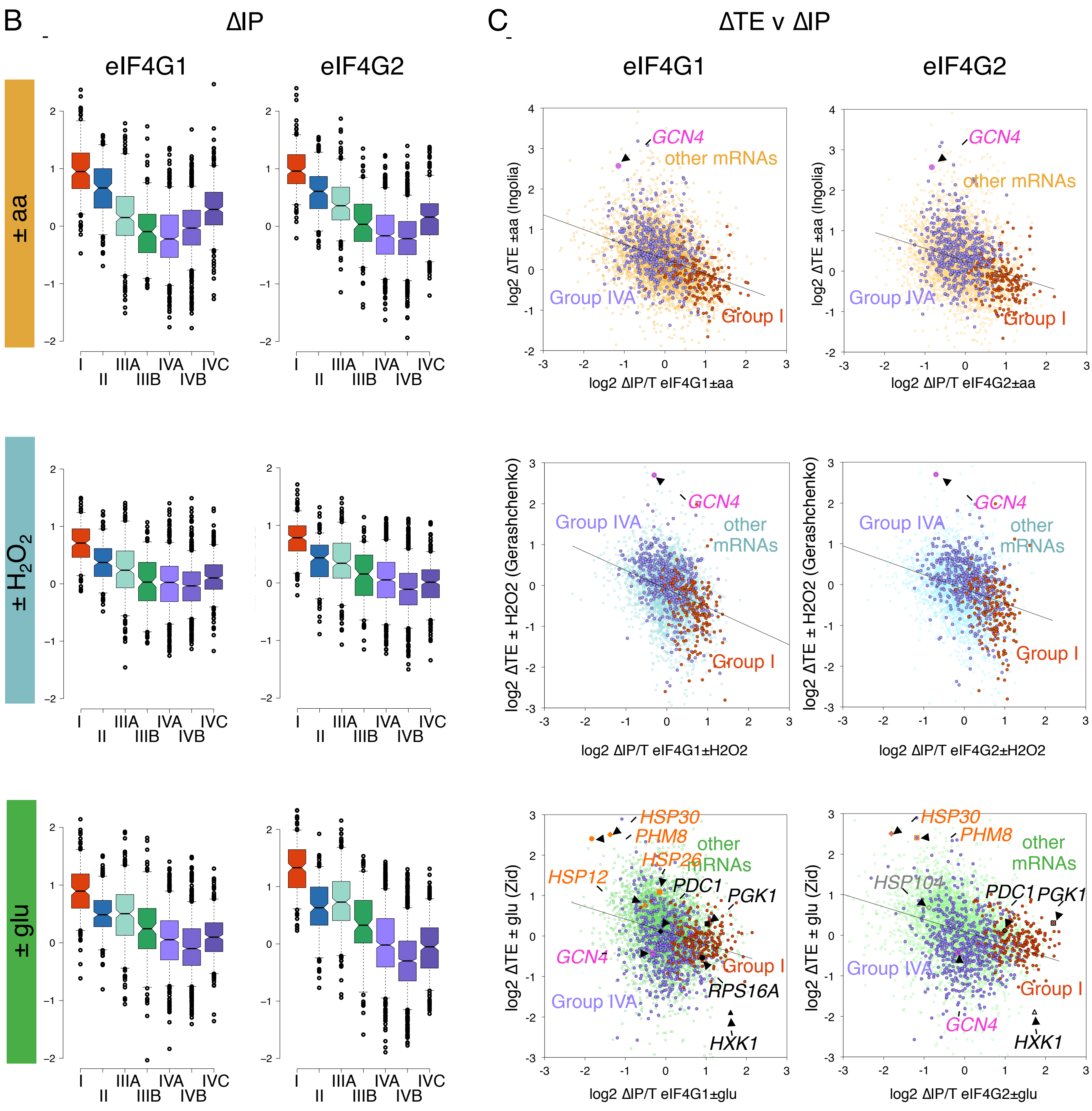

Supplement: Supplementary file 2 — Supplementary Figures. Figures and legends for Supplementary Figures S1–S6. (PDF 7144 kb) [file 13059_2017_1338_MOESM2_ESM.pdf]
